# A BLADE-ON-PETIOLE orthologue regulates corolla differentiation in the proximal region in *Torenia fournieri*

**DOI:** 10.1038/s41467-023-40399-3

**Published:** 2023-08-08

**Authors:** Shihao Su, Yawen Lei, Xuan Zhou, Takamasa Suzuki, Wei Xiao, Tetsuya Higashiyama

**Affiliations:** 1 School of Agriculture, Sun Yat-sen University, 518107 Shenzhen, China; 2 Institute of Nanfan & Seed Industry, Guangdong Academy of Science, 510316 Guangzhou, Guangdong China; 3 College of Bioscience and Biotechnology, Chubu University, Kasugai, Aichi 487-8501 Japan; 4 MBP-Center for Plant Molecular Biology, University of Tübingen, Auf der Morgenstelle 32, 72076 Tübingen, Germany; 5 Department of Biological Sciences, Graduate School of Science, The University of Tokyo, Tokyo, 113-0033 Japan; 6 Institute of Transformative Bio-Molecules (WPI-ITbM), Nagoya University, Furo-cho, Chikusa-ku, Nagoya, Aichi 464-8601 Japan

**Keywords:** Patterning, Plant morphogenesis, Pattern formation

## Abstract

The three-dimensional shape of a flower is integrated by morphogenesis along different axes. Differentiation along the petal proximodistal axis is tightly linked to the specification of pollinators; however, it is still unclear how a petal patterns this axis. The corolla of *Torenia fournieri* exhibits strong differentiation along the proximodistal axis, and we previously found a proximal regulator, TfALOG3, controlling corolla neck differentiation. Here, we report another gene, *TfBOP2*, which is predominantly expressed in the proximal region of the corolla. *TfBOP2* mutants have shorter proximal corolla tubes and longer distal lobe, demonstrating its function as a proximal regulator. *Arabidopsis BOP*s mutant shows similar defects, favouring a shared role of BOPs homologues. Genetic analysis demonstrates the interaction between *TfBOP2* and *TfALOG3*, and we further found that TfALOG3 physically interacts with TfBOP2 and can recruit TfBOP2 to the nuclear region. Our study favours a hypothetical shared BOP-ALOG complex that is recruited to regulate corolla differentiation in the proximal region axis of *T. fournieri*.

## Abstract (web summary)

Flowers manifest enormous diversity and shape is determined by morphogenesis along different independent axes. Here Su *et al*. report a shared BOP-ALOG module that regulates corolla differentiation in the proximal region axis.

## Introduction

Floral organs are hypothesized to have evolved from the ground state of leaf-like organs^1^. The final floral shapes of angiosperms vary considerably; this morphological diversity can be achieved in multiple ways, including changing floral symmetry, altering organ number or size, elaborating individual organs, and causing organ fusion^2,3^. The specific shape of a flower is a product of a long history of plant–pollinator interactions^4^. For example, the formation of corolla tubes (fusion of petals) in most asterid lineages has been regarded as a morphological innovation, which is possibly driven by animal pollinators and basically distinguishes asterids from rosids^5^.

The three-dimensional shape of a flower is integrated through differentiation in an organized fashion along different axes, such as the radial, dorsoventral and proximodistal axes. Floral radial and dorsoventral axes are established de novo within the plane of the floral meristem: the radial axis defines four concentric whorls of floral organs (i.e., sepal, petal, stamen and carpel) from the outermost to the central parts of a floral meristem, while the dorsoventral axis describes the relationship of different organs within one whorl (namely, dorsal, lateral and ventral). Several ancient modular programs have been discovered to explain the conserved developmental organogenesis of flowers along these two axes in various species^2,6^. According to the ABC model, the identities of four concentric floral organs along the radial axis are controlled by three classes of homeotic genes: A genes are required for the identities of sepals and petals, B genes are essential for petals and stamens, and C genes confer the identities of stamens and carpels^7^. Variations in organ identity are generally the result of changes in these organ identity genes, such as the petaloid bract of *Cornus florida* or the specification of lip in orchid flowers^8–10^. Differentiation along the dorsoventral axis results from the combined roles of TCP and MYB transcription factors: specifically, CYCLOIDEA-like (CYC-like) TCP protein and RADIALIS-like (RAD-like) MYB protein act together as dorsal factors, while another DIVARICATA-like (DIV-like) MYB protein provides a ventralizing signal^2,11–13^.

The proximodistal axis of a floral organ is defined by the polarized growth of organ primordia, which initiate away from the plane of the floral meristem and are elaborated by different cell and tissue types^14^. In terms of the petal, a billboard organ of an insect-pollinated flower that attracts its pollinators, differentiation along the proximodistal axis is evident in the pigmentation pattern, epidermal micromorphology or specification of the petal surface, and such differentiation becomes a major component of floral diversity in nature. In Arabidopsis, *JAGGED* (*JAG*) encodes a zinc finger transcription factor, promoting distal growth of leaves and petals, as the *jag* mutant possesses serrated distal organ margins^15,16^. The *cin* (*cincinnata*) mutant from *Antirrhinum majus* has larger leaves due to excess growth of leaf margins and smaller petal lobes that are correlated with specific expression of *CIN* in distal corolla lobes, favouring its roles in regulating growth and differentiation of both leaves and petals^17^. Despite these findings, factors specifically involved in patterning the proximodistal axis of floral organs have yet to be identified and most downstream regulators of petal cell differentiation also remain to be isolated.

The genetic regulation of cell differentiation along the proximodistal axis of plant lateral organs is mainly studied using leaves as a model. *Class I KNOTTED* homeobox (*KNOX I*) genes, which promote indeterminate growth, are expressed in meristematic tissues^14^. Mutant analyses indicate that ectopic expression of *KNOX I* genes in leaves results in changes in leaf proximodistal patterning, as in gain-of-function maize mutants^18^. A typical leaf of eudicots can be subdivided into two regions, including a proximal petiole and a distal blade. In Arabidopsis, *bop1* (*blade on petiole1*) and *bop2* (*blade on petiole2*) mutants have ectopic lobed blades along the petiole, suggesting that BOP transcription factors suppress blade outgrowth and are indispensable for the growth of the proximal region of a leaf^19–21^. In addition, BOPs posttranscriptionally regulate LEAFY (LFY) activity, promoting flower development by forming an adaptor with LFY and CULLIN3 (CUL3)-RING ubiquitin ligases^22^. In legume species, including pea and *Lotus japonicus*, *BOP* genes are involved in the differentiation of stipules and leaflet-like organs in the proximal part of leaves^23,24^. In monocots, such as rice and barley, the proximal region of a leaf is called the sheath. The monocot *BOP* genes show strong expression in leaf primordia as well as the proximal sheath margins, and mutant analyses indicate that they are essential for development of the proper sheath-blade ratio by promoting sheath differentiation and suppressing blade development^25,26^. Furthermore, BOP proteins are also involved in many other biological processes, including organ boundary formation, plant architecture establishment, nodule development and abscission zone formation^27^. However, whether BOPs are involved in petal proximodistal differentiation and whether they have a role in the formation of corolla tubes are still unknown.

To investigate the molecular mechanisms involved in corolla proximodistal differentiation, we studied the wishbone flower, *Torenia fournieri* (Linderniaceae), and we previously divided the floral developmental process of *T. fournieri* into 14 stages based on floral organ morphologies^11^. The corolla of a mature *T. fournieri* flower can be divided into several subregions due to the presence of different pigmentations along its proximodistal axis (Supplementary Fig. 1). The distal part of petals forms a lobe region, in which separated lateral and ventral petal lobes have violet pigmented spots; a middle corolla region forms a conical tube, which itself can be further subdivided into a distal part with light purple pigments and a proximal part with abundant yellow carotenoids; and a corolla base that is specified into an inflated neck region, which may help flowers to prevent raindrops from contacting the nectar inside the corolla (Supplementary Fig. 1). We recently reported that *TfALOG3*, an *ALOG* (*Arabidopsis LSH1* and *Oryza G1*) family gene, is expressed in the epidermal cells of the proximal corolla neck, and *TfALOG3* loss-of-function mutants fail to differentiate this part of the organ^28^. Since ALOG proteins in tomato and pea have been shown to interact with BOP proteins, we propose the existence of a conserved protein dimer formed by BOP and ALOG within plants^29–31^. Here, we isolated a floral-specific *BOP* gene from *T. fournieri*, *TfBOP2*, which is predominantly expressed in the proximal corolla regions. Loss-of-function mutants of *TfBOP2* show developmental defects in proximal corolla differentiation, which is correlated with its expression pattern. The function of BOP proteins in petal proximodistal differentiation is evolutionarily conserved in Arabidopsis. We also found genetic and physical interactions between TfALOG3 and TfBOP2, favouring our hypothesis of a shared interaction between ALOG and BOP across a subset of flowering plants, the core eudicots.

## Results

### *TfBOP2* is expressed predominantly in the proximal regions of the corolla

**Fig. 1:**
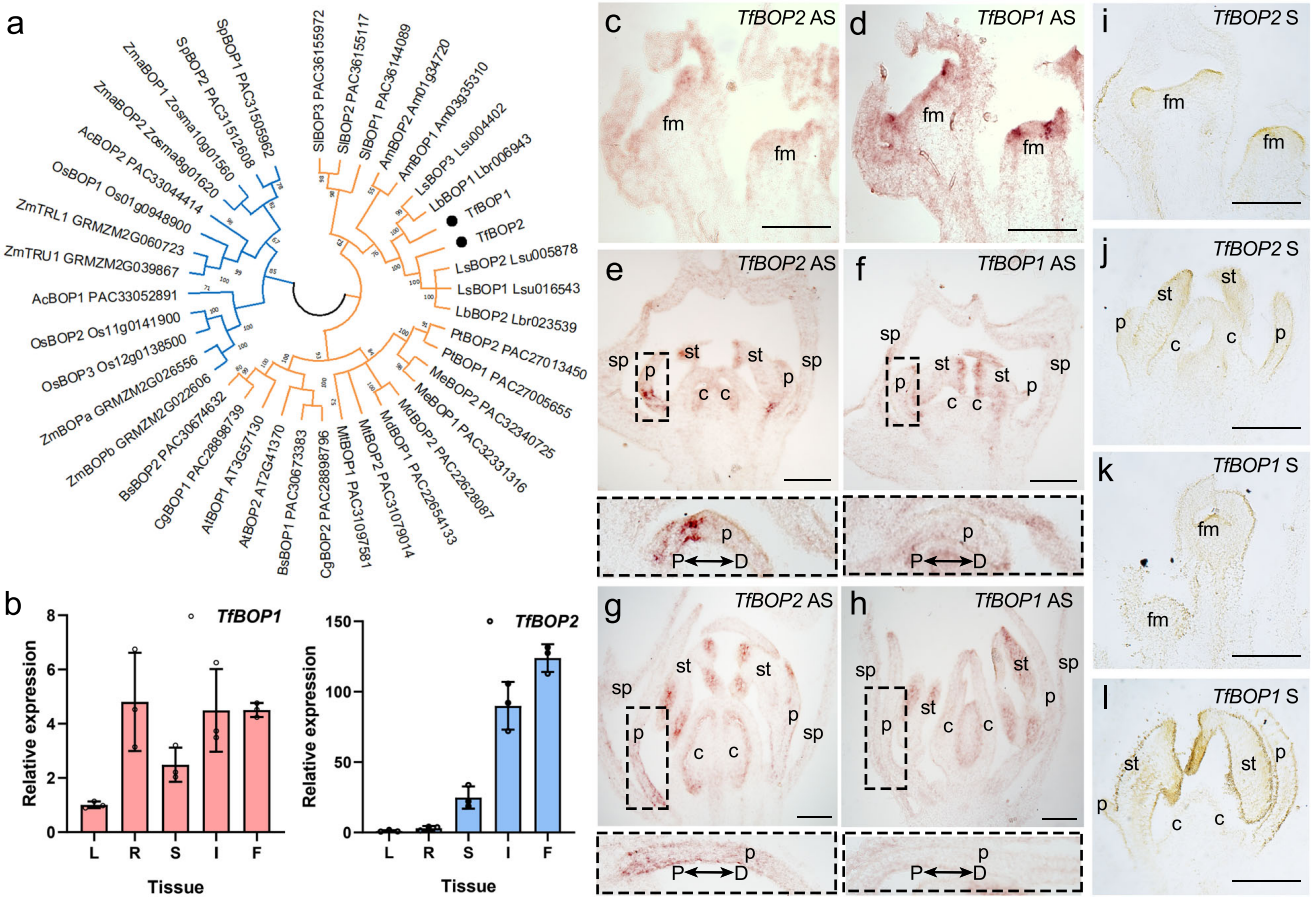
Phylogenetic and expression analysis of *BOP*s. **a** Maximum-likelihood tree of BOP-like proteins from *Spirodela polyrhiza* (Sp), *Ananas comosus* (*Ac*), *Zostera marina* (*Zma*), *Oryza sativa* (*Os*), *Zea mays* (*Zm*), *Solanum lycopersicum* (*Sl*), *Medicago truncutula* (*Mt*), *Malus domestica* (*Md*), *Populus trichocarpa* (*Pt*), *Manihot esculenta* (*Me*), *Arabidopsis thaliana* (*At*), *Boechera stricta* (*Bs*), *Capsella grandiflora* (*Cg*), *Antirrhinum majus* (*Am*), *Lindernia brevidens* (*Lb*), *Lindernia subracemosa* (*Ls*) and *Torenia fournieri* (*Tf*). Branches of monocot and core eudicot genes are highlighted in blue and orange, respectively; 500 bootstrap replicates (percentage) are marked at each node, and the accession number for each sequence is included as a suffix. **b** Quantitative reverse transcription polymerase chain reaction (qRT‒PCR) analysis of *TfBOP1* and *TfBOP2* in different tissues. *β-Actin* (*TfACT3*) was used as an internal control. R, root; L, leaf; S, stem; I, inflorescence; F, stage 10 flower bud. Data were normalized by the relative expression in leaf. Error bars of gene expression are SDs from three biological replicates. **c**–**l** RNA in situ hybridization of *TfBOP1* and *TfBOP2* in stage 0-1 (**c**, **d**, **i**, **k**), stage 6 (**e**, **f**, **j**, **l**) and stage 8 (**g**, **h**) flower buds of wild-type plants using antisense (AS) or sense (S) probes. Similar results were obtained from three independent experiments. fm floral meristem, sp sepal, p petal, st stamen, c carpel. Zoomed-in views of petals are marked with dashed boxes, where the petal proximal (P)–distal (D) axes are marked by black arrows; Scale bars: 100 μm.

We isolated two *BOP* genes from the genome of *T. fournieri*. Sequence alignments using TfBOP1/2 and Arabidopsis BOP1/2 proteins suggest three conserved domains in these proteins (Supplementary Fig. 2), including a BTB/POZ domain in the N-terminus, an ANK-repeat domain in the C-terminus, and an unknown domain DUF3420 that partially overlaps with the ANK-repeat domain. To understand the evolutionary history of BOP proteins, we carried out phylogenetic analysis by using BOP proteins from different plant lineages. The phylogenetic tree indicates that BOP proteins originated anciently and are distributed in various species (Fig. 1a). Multiple duplication events for BOP-like proteins occurred independently in different plant lineages, including monocots (*Spirodela polyrhiza* (Sp), *Ananas comosus* (*Ac*), *Zostera marina* (*Zma*), *Oryza sativa* (*Os*), *Zea mays* (*Zm*)) and core eudicots (*Solanum lycopersicum* (*Sl*), *Medicago truncutula* (*Mt*), *Malus domestica* (*Md*), *Populus trichocarpa* (*Pt*), *Manihot esculenta* (*Me*), *Arabidopsis thaliana* (*At*), *Boechera stricta* (*Bs*), *Capsella grandiflora* (*Cg*), *Antirrhinum majus* (*Am*), *Lindernia brevidens* (*Lb*), *Lindernia subracemosa* (*Ls*) and *Torenia fournieri* (*Tf*)). *Torenia* belongs to the family Linderniaceae, which contains two specific subclades of BOP proteins (Fig. 1a), signifying that a duplication event occurred prior to the divergence of the *Torenia* and *Lindernia* genera. We further wanted to know whether the two paralogues of Torenia BOP fulfil distinct function or act redundantly.

We examined the expression patterns of two *TfBOP*s using quantitative reverse transcription (qRT)-PCR and RNA in situ hybridization assays. qRT‒PCR showed that *TfBOP1* is expressed in various tissues, with high expression levels in roots, stems, inflorescences and stage 10 flowers; however, *TfBOP2* is preferentially expressed in reproductive tissues such as inflorescences and stage 10 flowers, suggesting functional diversification of these two paralogues (Fig. 1b). RNA in situ hybridization assays demonstrate that *TfBOP1* is strongly expressed in the early stages of floral meristems, whereas *TfBOP2* is not (Fig. 1c, d). In stage 6 (Fig. 1e, f) and stage 8 (Fig. 1g, h), both genes are highly expressed in the stamens and ovules; however, they show divergent expression patterns in the corolla. Compared with the controls using sense probes (Fig. 1i–l and Supplementary Fig. 3), signals using antisense probes of *TfBOP1* are dispersed in the whole corolla, while *TfBOP2* antisense probes show strong signals in the proximal region of the corolla. This specific expression of *TfBOP2* suggests a role in cell differentiation in the proximal petal region.

### TfBOP2 is necessary for correct differentiation of the corolla epidermis

We then explored the function of TfBOP2 by obtaining *TfBOP2* loss-of-function mutants. A CRISPR/Cas9 plasmid containing three *TfBOP2*-specific guide RNAs was constructed and transformed into *T. fournieri*. To avoid off-target effects, the candidate target sequences were queried against the reference genome using BLASTN, ensuring at least two bases different from any similar nontarget sequences within the protospacer-adjacent motif (PAM) proximal region. Seven hygromycin-resistant lines with similar floral phenotypes were obtained, and the *TfBOP2* genes from two independent lines (hereafter called, *tfbop2-2* and *tfbop2-5*) were sequenced prior to further analysis (Supplementary Fig. 4). We detected different numbers of nucleotide deletions or insertions in the targeted sites of the *TfBOP2* gene, leading to frame shift and premature termination (Supplementary Fig. 4). There was no difference within the *TfBOP1* genes among the two lines, supporting the specificity of editing.

**Fig. 2:**
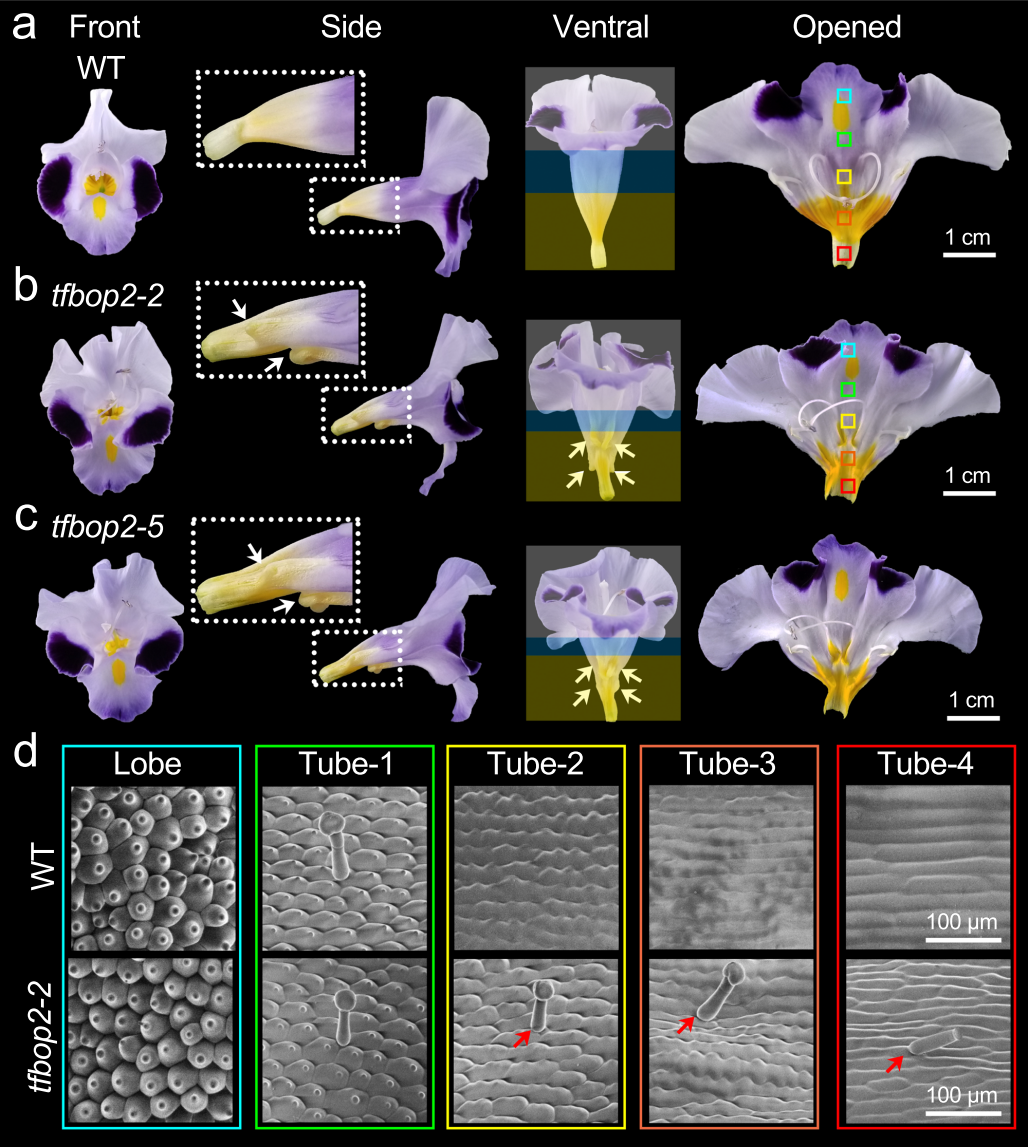
Floral morphologies of wild-type (WT) and *TfBOP2*-Cas9 mutants. **a**–**c** Front, side, ventral and opened views of WT (**a**) and two independent *TfBOP2*-Cas9 lines: *tfbop2-2* (**b**) and *tfbop2-5* (**c**). Yellow, blue and white blocks indicate the proximal tube, distal tube and lobe regions, respectively. White arrows indicate ectopic tissues in the corollas of *TfBOP2*-Cas9 lines. Zoomed-in view of proximal corolla are marked with white dashed boxes. Different colour boxes represent regions for observing epidermal cells. **d** Corolla epidermal cell morphologies of WT (upper panel) and the *tfbop2-2* mutant (lower panel); whole corollas are divided into five regions along the proximodistal axis (Lobe, Tube 1/2/3/4) marked by different colours; red arrows indicate ectopic trichomes on the corollas of *tfbop2-2* plants. Similar results were obtained from three biological replicates.

We later compared the floral morphologies of the wild-type (WT) and *TfBOP2*-Cas9 plants (Fig. 2). In *tfbop2-2* and *tfbop2-5*, developmental defects of the proximal corolla region were detected, including the proximal tube and corolla neck (Fig. 2a–c). In the WT, yellow pigments preferentially accumulated in the proximal region of the corolla tube; however, these pigments became light and ectopically shifted to the corolla bottoms of *TfBOP2*-Cas9 plants (Fig. 2a–c). Second, *TfBOP2*-Cas9 flowers often show abnormalities in corolla tube fusion. Compared with the flowers of the WT, the flowers of *TfBOP2*-Cas9 plants have distorted corolla forms from the front view (Fig. 2a–c). Moreover, cells in the corolla fusion boundaries became excessively proliferative, as we found that ectopic tissues developed in these regions (Fig. 2b, c).

Since epidermal cell morphologies vary along the petal proximodistal axis, we then compared the corolla epidermis using scanning electron microscopy. In the WT plant, we divided the corolla into five subregions along the proximodistal axis, including one lobe region (Lobe) and four types of tube regions (Tube-1–Tube-4) (Fig. 2d). Conical cells, flat conical cells, puzzle-shaped cells with wavy edges, puzzle-shaped cells with smooth edges and rectangular cells are the representative cell types of the five regions (Fig. 2d). In addition, trichomes are found specifically in the distal Tube-1 region of WT corollas (Fig. 2d). Compared with WT, only the epidermal cells from the proximal regions of the corolla were affected in the *tfbop2-2* mutant (Fig. 2d). Trichomes are ectopically found in all regions of mutant corollas, including the Tube-3 and Tube-4 regions. Second, epidermal cells of the Tube-2 region are smaller, while those of the Tube-3 region are puzzle-shaped cells with wavy edges, similar to those of the WT Tube-2 region. In addition, epidermal cells of the Tube-4 region are smaller than those of the WT region (Fig. 2d, see below Fig. 6f). These results demonstrate that TfBOP2 controls proximal corolla differentiation by acting on cell expansion.

### TfBOP2 controls the corolla lobe–tube ratio

**Fig. 3:**
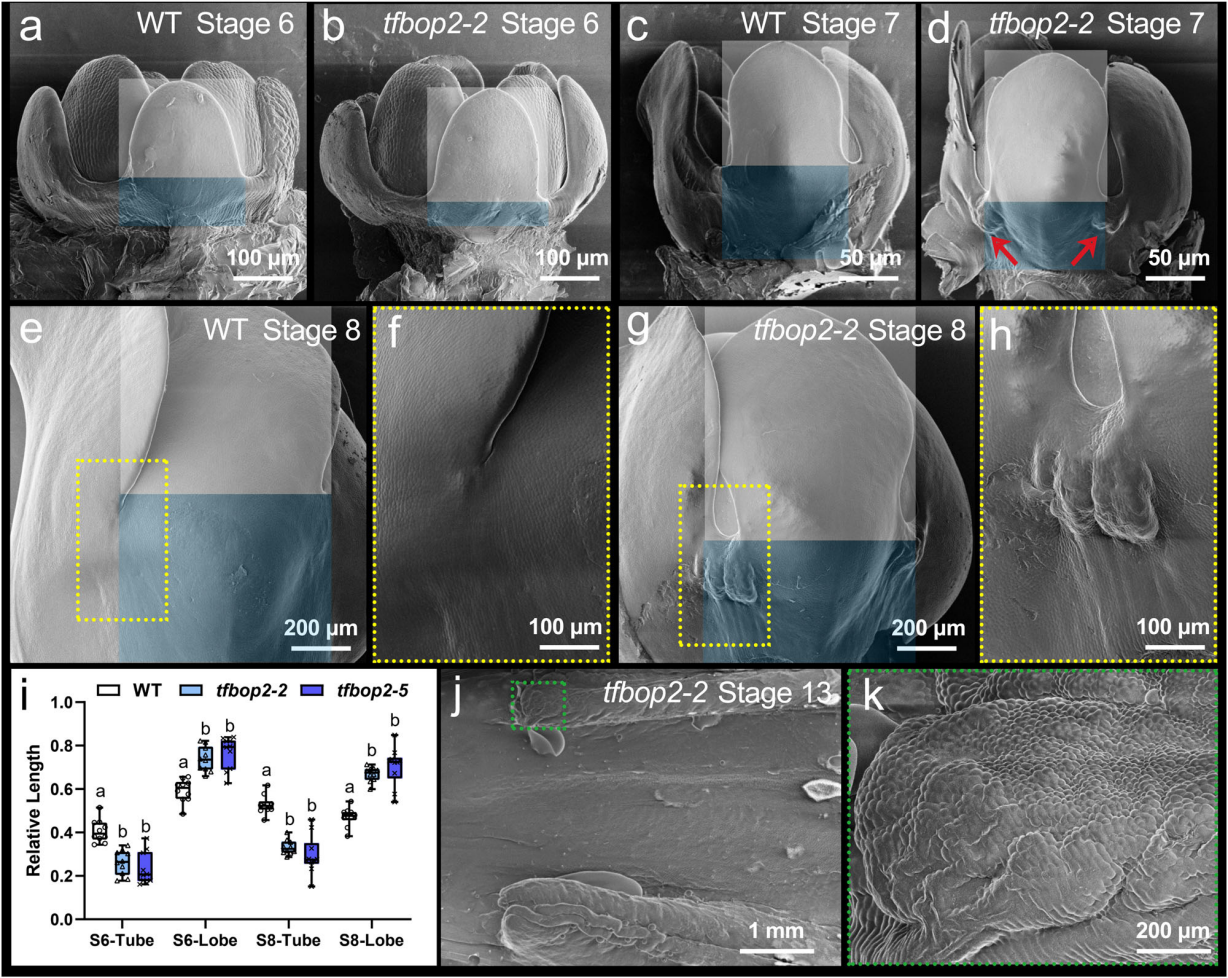
Floral organogenesis of wild-type (WT) and *tfbop2-2* plants observed by scanning electron microscopy. **a**, **b** Stage 6 flowers of WT and *tfbop2-2*. **c**, **d** Stage 7 flowers of WT and *tfbop2-2*. **e**–**h** Stage 8 flowers of WT and *tfbop2-2*; yellow dashed boxes mark zoomed-in views of the fusion margins of *tfbop2-2*. **i** Relative corolla lengths (Lengths of tube or lobe region/lengths of petal) of WT and two *TfBOP2*-Cas9 mutants. S6: Stage 6; S8: Stage 8. Letters indicate significant differences determined by two-way ANOVA with Tukey’s multiple comparisons test (two-sided, *P* < 0.05); Boxplots display quartiles Q1, Q2 (centre), and Q3, with whiskers extending to the furthest data point from 10 flower buds; each *P* value adjusted to account for multiple comparisons is shown in the source data file. **j** A stage 13 flower of *tfbop2-2*. **k** Green dashed boxes mark zoomed-in views of ectopic tissues in the fusion margins of *tfbop2-2*. Blue and white blocks indicate the tube and lobe regions, respectively; red arrows indicate ectopic tissues in the fusion margins of *tfbop2-2*. Similar results were obtained from three biological replicates.

To understand the developmental defects of *TfBOP2*-Cas9 flowers, we compared floral organogenesis of WT and *tfbop2-2/5* under scanning electron microscopy (Fig. 3 and Supplementary Fig. 5). In stage 6, stamens are larger than petals. General floral morphologies of WT and *tfbop2-2/5* seem similar; however, the proximal tube domain within mutants is reduced and the lobe domain is increased relative to the wild type. Increased lobe–tube ratios were also found in stage 8 (Fig. 3i). Since *TfBOP2* is not normally expressed in the petal lobe, the increased distal region in the *tfbop2* mutant could be an indirect effect resulting from a compensation mechanism to maintain overall petal length: in absence of TfBOP2 activity, the proximal region grows less and this feedbacks on the overall control of growth, indirectly promoting growth of the distal region.

As mentioned before, *TfBOP2-*Cas9 flowers also exhibit developmental abnormities in corolla tube formation. We observed these defects starting from stage 7 (Fig. 3c, d and Supplementary Fig. 5). We found enlarged tissues formed in the fusion margins of mutant corolla tubes, and these tissues further proliferated along with the development of flowers (Fig. 3e–h and Supplementary Fig. 5). We also noticed that the epidermal cells on these ectopic tissues were small and spherical and were morphologically similar to epidermal cells in distal lobe regions (Figs. 3j, k and 2d). These findings favour the hypothesis that loss of TfBOP2 results in disrupted differentiation of the proximal corolla.

### TfBOP2 regulates cell differentiation in the proximal region possibly by targeting genes related to meristem and boundary formation

**Fig. 4:**
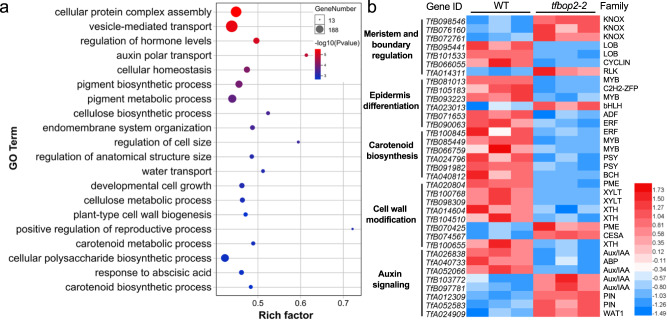
Downstream pathways and candidate genes possibly regulated by TfBOP2. **a** Differentially enriched Gene Ontology (GO) terms between the corollas of wild-type (WT) and *tfbop2-2* constructed by Omicshare (https://www.omicshare.com/). Bubble size and colour represent gene number and *P* value (two-sided, FDR-adjusted), respectively. Rich factor is defined as the ratio of input DEGs that are annotated in a GO term to all genes that are annotated in this GO term. **b** Heatmap of candidate downstream genes from three biological replicates of transcriptomic data generated by Heml (https://hemi.biocuckoo.org). Red and blue colours represent upregulated and downregulated transcripts, respectively.

To investigate how TfBOP2 is involved in corolla differentiation, we carried out transcriptome sequencing using total RNA from stage 10 corollas. We further performed differential gene expression analysis between WT and *tfbop2-2* using edgeR (Supplementary Data 1). There were 435 significantly upregulated and 788 significantly downregulated contigs in the *tfbop2-2* mutant (Adjusted *P* value < 0.0001, |LogFC | > 1). Gene Ontology (GO) enrichment was performed using the contigs that were significantly changed in the *tfbop2-2* mutant (Supplementary Data 2). Among the significantly enriched GO terms (*P* < 0.05), terms annotated as protein complex assembly, auxin polar transport, pigment biosynthesis, cell wall metabolism, regulation of anatomical structure and developmental cell growth were included (Fig. 4a). The differentially expressed genes (DEGs) contain many candidates acting as potential downstream targets of TfBOP2, including factors involved in meristem and boundary formation, epidermis differentiation, carotenoid biosynthesis, cell wall modification, and auxin signalling (Fig. 4b and Supplementary Data 3). These GO terms and DEGs are correlated with the morphological changes in *tfbop2-2* flowers. Among the 7 DEGs related to meristem and boundary formation, three *KNOX I* genes (*TfB098546*, *TfB076160* and *TfB072761*) were significantly upregulated in the *tfbop2-2* mutant (Fig. 4b). *TfB098546* (*TfKNAT2*) and *TfB076160* (*TfKNAT6*) are orthologues of Arabidopsis *KNAT2/6*, while *TfB072761* is an orthologue of Arabidopsis *STM* (Supplementary Fig. 6). We further performed RNA in situ hybridization assays to detect gene expression patterns of two *KNOX I* genes in the stage 8 flowers of WT and *tfbop2-2* mutant. Our results show that both *TfKNAT2* and *TfKNAT6* are ectopically expressed in the proximal petal region of *tfbop2-2* mutant (Supplementary Fig. 6). Hence, we inferred that TfBOP2 may be able to restrict the expression of *KNOX I* genes at the flower base, thus coordinating corolla tube fusion along the proximodistal axis.

### The function of BOPs in cell differentiation at the petal base is conserved in Arabidopsis

**Fig. 5:**
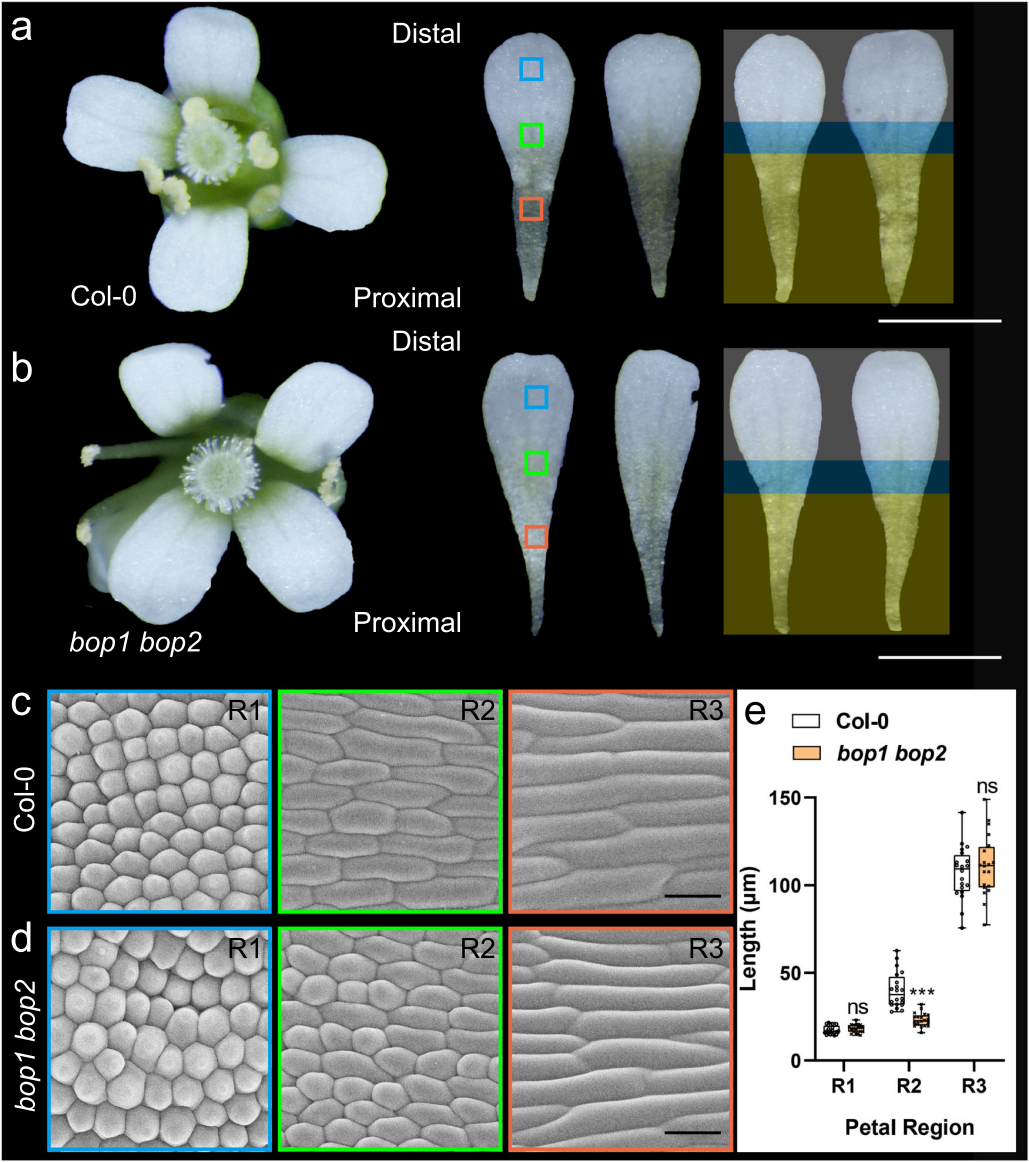
Floral morphologies of wild-type (Col-0) and *bop1 bop2* mutant *Arabidopsis thaliana*. **a**, **b** Intact flowers and dissected petals of WT (**a**) and *bop1 bop2* mutant (**b**). Yellow, blue and white blocks indicate the proximal claw, intermediate and distal blade regions, respectively; Scale bars: 1 mm. **c**, **d** Corolla epidermal cell morphologies of WT (**c**) and *bop1 bop2* mutant (**d**). Whole petals are divided into three regions along the proximodistal axis (R1/2/3) marked by different colours. Scale bars: 25 μm. **e** Epidermal cell length of the R1–R3 regions of corolla tubes in the WT and mutant. Significant differences are determined by two-way ANOVA with Tukey’s multiple comparisons test (two-sided, ***P < 0.0001); ns represents no significant difference. Boxplots display quartiles Q1, Q2 (centre), and Q3, with whiskers extending to the furthest data point from 20 epidermal cells; each *P* value adjusted to account for multiple comparisons is shown in the Source data file.

To understand whether the function of BOPs in petal cell differentiation is conserved, we examined the flower morphologies of the WT and the *bop1 bop2* mutant of Arabidopsis (Fig. 5 and Supplementary Fig. 7). Although Arabidopsis does not have a fused corolla tube, cell differentiation also manifests along the proximodistal axis, as a WT Arabidopsis petal has a white distal blade and a greenish proximal claw (Fig. 5a). In the *bop1 bop2* mutant, however, the white blade regions become longer, while the proximal claws are sharper (Fig. 5b). We further compared the petal epidermal cells of these flowers. Epidermal cells were generally similar in the R1 and R3 regions, with no significant difference in epidermal cell length (Fig. 5c–e). However, the average cell length became significantly shorter in the R2 region of the *bop1 bop2* mutant, and we found ectopic conical cells in this region, suggesting an elongated blade region (Fig. 5c–e and Supplementary Fig. 7). Together with our findings in *T. fournieri*, we proposed that the function of BOPs in regulating cell differentiation in the petal proximal region is shared across lineages.

### *TfBOP2* genetically interacts with *TfALOG3* to control proximal cell differentiation of the corolla

**Fig. 6:**
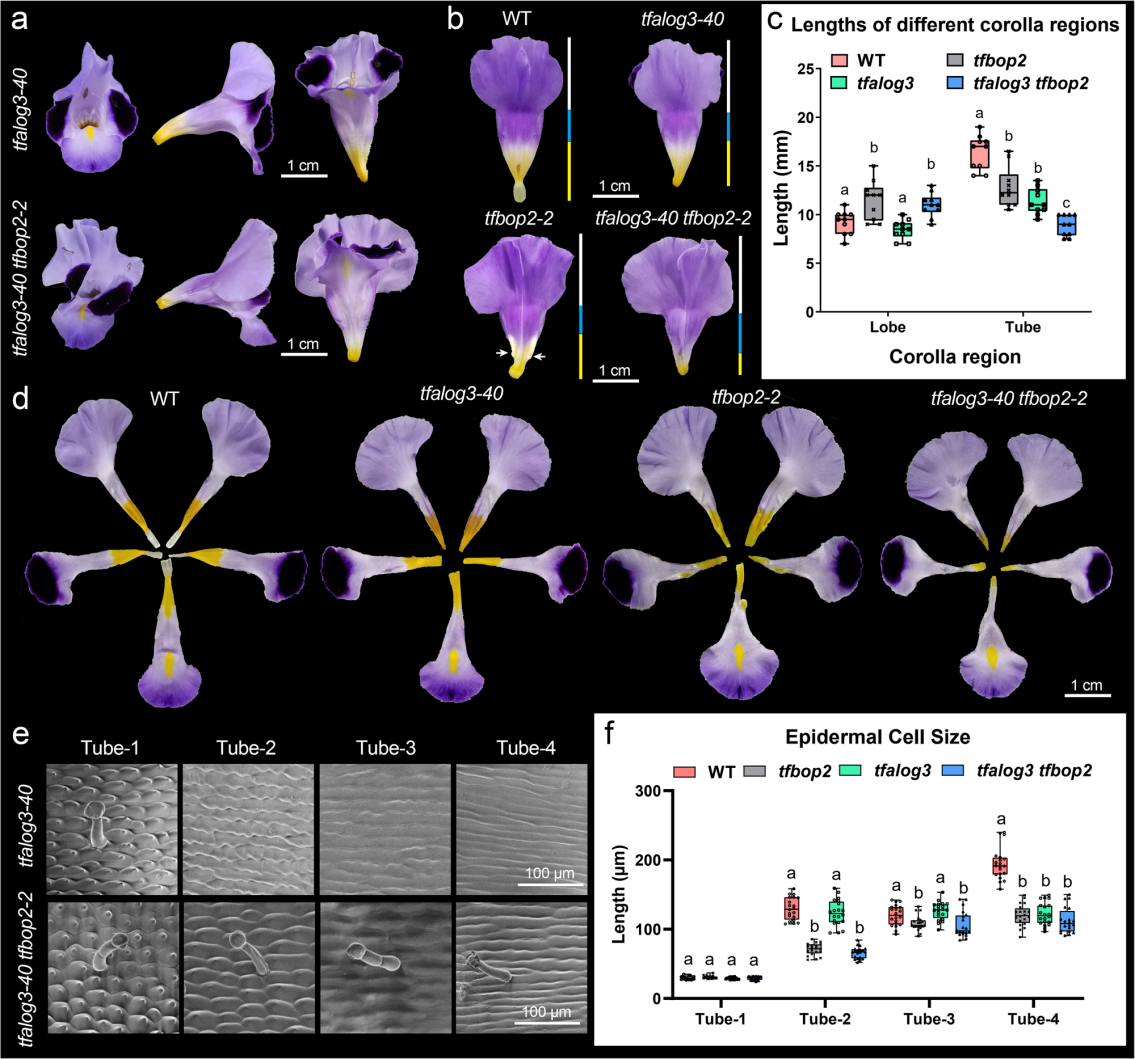
Genetic and physical interactions between TfBOP2 and TfALOG3. **a** Front, side and ventral views of *tfalog3-40* and *tfalog3-40 tfbop2-2* mutants. **b** Dorsal views of corollas of different genotypes; yellow, blue and white bars represent the lengths of the proximal tube, distal tube and lobe regions, respectively. White arrows indicate ectopic tissues in *tfbop2-2*. **c** Lengths of different corolla regions in the WT and mutants; letters indicate significant differences determined by two-way ANOVA with Tukey’s multiple comparisons test (two-sided, *P* < 0.05). Boxplots display quartiles Q1, Q2 (centre), and Q3, with whiskers extending to the furthest data point from 10 flowers; each *P* value adjusted to account for multiple comparisons is shown in the source data file. **d** Five petals dissected from typical flowers of different genotypes. **e** Corolla epidermal cell morphologies of *tfalog3-40* and *tfalog3-40 tfbop2-2* mutants; T1-T4 represent four regions from distal to proximal regions of corolla tubes. **f** Epidermal cell length of the T1-T4 regions of corolla tubes in the WT and mutants; letters indicate significant differences determined by two-way ANOVA with Tukey’s multiple comparisons test (two-sided, *P* < 0.05). Boxplots display quartiles Q1, Q2 (centre), and Q3, with whiskers extending to the furthest data point from 20 epidermal cells; each *P* value adjusted to account for multiple comparisons is shown in the Source data file.

The flower of *T. fournieri* is facing upward and raindrops easily enter the flower, we previously reported that flowers of *TfALOG3-*Cas9 failed to develop into a proximal corolla neck and cannot prevent water from entering the nectary^28^. To investigate whether the flowers of *TfALOG3* and *TfBOP2* loss-of-function mutants share similar defects, we compared their floral morphologies and validated the water-repellent property. By applying amount of water containing 0.1% Safranin O dye as an indicator into different corolla tubes, we observed coloured water entered the nectary region of both *tfalog3-40* and *tfbop2-2* flowers (Supplementary Fig. 8). In the WT, a clear boundary is observed in the constriction region with hairs that connect the proximal yellow tube and corolla neck; however, this boundary is no longer apparent in *tfalog3-40* or *tfbop2-2* flowers (Supplementary Fig. 9). Although both *tfalog3-40* and *tfbop2-2* share similar developmental defects within the proximal corolla, their proximal corolla tube regions are not identical: the corolla neck part is completely lost in *tfalog3-40* but not in *tfbop2-2*, while ectopic tissues developed in the corolla fusion boundaries of *tfbop2-2* cells are not observed in *tfalog3-40* (Figs. 2b and 6a, b). *TfALOG3* and *TfBOP2* have overlapped expression patterns in the proximal corollas, indicating potential genetic interaction between them. Hence, we further investigated the morphology of the *TfALOG3* and *TfBOP2* double mutant.

Similar to *tfbop2-2*, the *tfalog3-40 tfbop2-2* double mutant also possesses a distorted corolla (Figs. 2b and 6a, b). Interestingly, unlike *tfbop2-2*, the defect in corolla fusion was largely rescued, as we could not find ectopically developed tissues at the fusion margins, suggesting that TfALOG3 may antagonize TfBOP2’s activity in corolla boundary (Fig. 6a, b). In addition, we noticed that the carotenoid-enriched tube region in *tfalog3-40 tfbop2-2* was strikingly shortened compared with that in either *tfalog3-40* or *tfbop2-2*, indicating that TfALOG3 functions together with TfBOP2 in proximal corolla differentiation (Fig. 6b–d). We further measured the lengths of different corolla regions among the four genotypes (Fig. 6c). The length of the lobe region does not change in the *tfalog3-40* mutant but becomes significantly longer in *tfbop2-2* or *tfalog3-40 tfbop2-2* double mutants. In terms of the length of the tube region, the single mutant of either *tfbop2-2* or *tfalog3-40* was significantly shorter than the WT, and the double mutant had the shortest corolla tube (Fig. 6c).

As corolla proximodistal differentiation is also evident in epidermal cell morphology, we examined the corolla epidermal cells in single and double mutants (Fig. 6e, f). Five typical types of epidermal cells are also found in *tfalog3-40*, and compared with WT, only rectangular cells in the Tube-4 region decrease, consistent with our previous findings^28^. In either *tfbop2-2* or *tfalog3-40 tfbop2-2*, epidermal cells in the Tube-2/3/4 regions were significantly smaller than those in the WT, and we observed ectopic trichomes in these regions of the corolla tubes, suggesting that TfALOG3 works together with TfBOP2 through control of proximal cell expansion as cell differentiate (Figs. 2d and 6e). Altogether, these results favour genetic interaction between *TfALOG3* and *TfBOP2*, as double mutants have the shortest proximal corolla tube while exhibiting rescue of *tfbop2-2*’s boundary defects.

### TfBOP2 physically interacts with TfALOG3

**Fig. 7:**
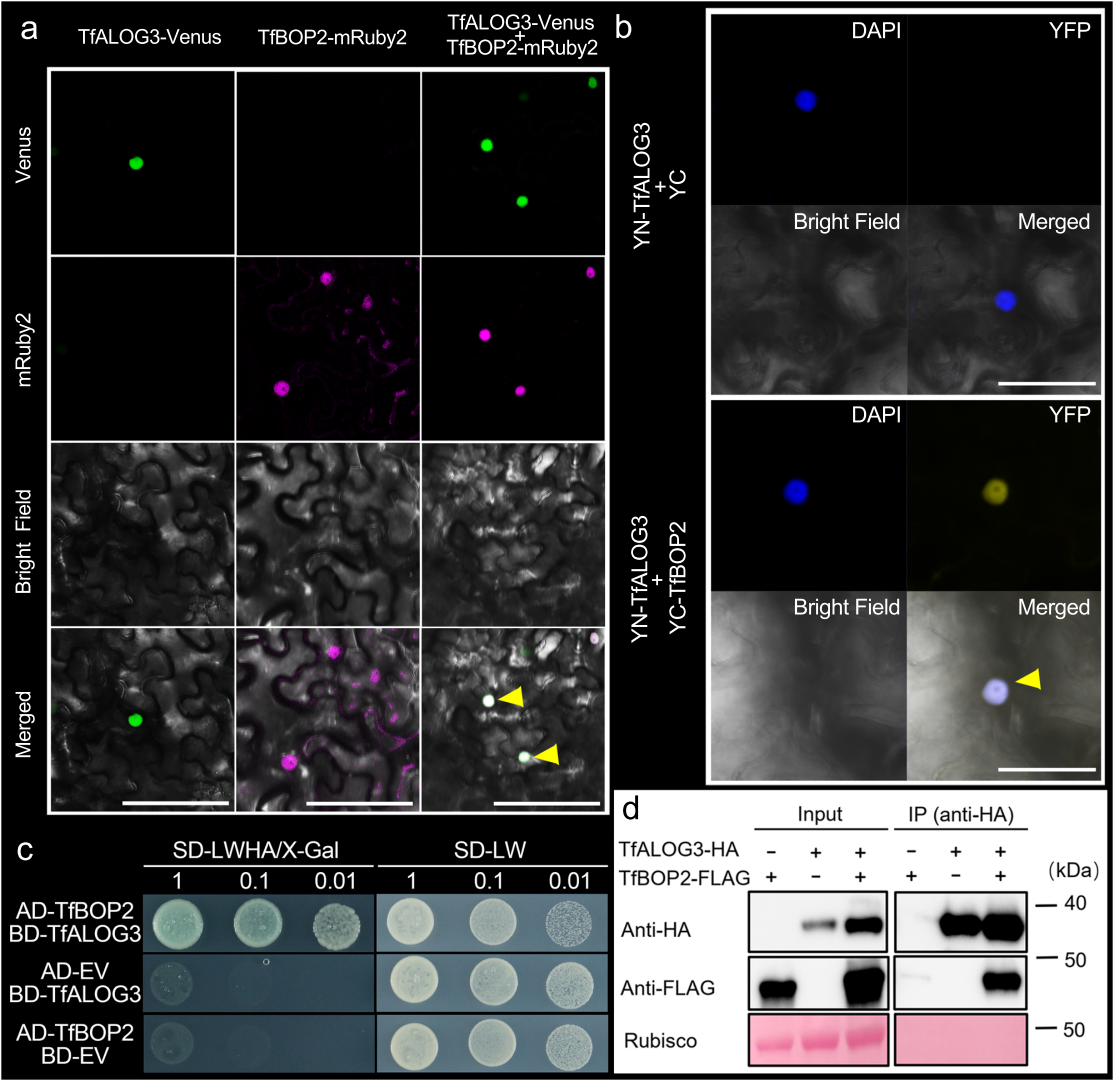
Physical interaction between TfBOP2 and TfALOG3. **a** Subcellular localization of transiently expressed TfALOG3-Venus or/and TfBOP2-mRuby2 fused proteins in tobacco leaves; Yellow arrows indicate co-localization of two proteins. Scale bars: 100 μm. TfALOG3 was previously shown to localize to the nucleus using Arabidopsis mesophyll protoplasts^32^. **b** Bimolecular fluorescence complementation (BiFC) assays in tobacco leaves showing that TfBOP2 interacts with TfALOG3 in the nucleus indicated by a yellow arrow. Scale bars: 50 μm. **c** Yeast two-hybrid assays showing that TfBOP2 interacts with TfALOG3. AD and BD represent the pGADT7 and pGBKT7 vectors, respectively; SD-LWHA/X-Gal and SD-LW indicate selective (SD medium minus Trp, Leu, His, Ade and supplemented with X-α-Gal) and nonselective (SD minus Trp and Leu) media. **d** Coimmunoprecipitation showing the interaction of TfBOP2 with TfALOG3. TfALOG3-HA was coimmunoprecipitated with anti-HA beads from tobacco leaves that coexpressed TfBOP2-FLAG and TfALOG3-HA. Ponceau S staining of Rubisco is shown as a loading control. Similar results were obtained from three independent experiments.

We further examined the subcellular localization of TfALOG3 and TfBOP2 by transient expression of fused proteins in tobacco leaf epidermal cells (Fig. 7a). We found that TfALOG3-Venus proteins are located in the nucleus, which is consistent with our previous data using Arabidopsis mesophyll protoplasts^32^. Unlike TfALOG3-Venus, transiently expressed TfBOP2-mRuby2 proteins showed both cytoplasmic and nuclear localization (Fig. 7a). We next wanted to determine whether the localization of TfBOP2 proteins can be affected by the coexpression of TfALOG3. The results showed that TfBOP2-mRuby2 signals exclusively shifted to the nuclear region when *TfALOG3* was cotransformed, suggesting that TfBOP2 can be recruited to the nucleus by TfALOG3, possibly by forming a transcriptional complex (Fig. 7a).

To verify this hypothesis, we performed bimolecular fluorescence complementation (BiFC), yeast two hybrid (Y2H) and coimmunoprecipitation (CoIP) assays (Fig. 7b–d). BiFC assays confirmed that TfBOP2 interacts with TfALOG3 in the nucleus (Fig. 7b). We also demonstrated that full-length TfBOP2 proteins interact with TfALOG3 in yeast (Fig. 7c). CoIP assays in tobacco leaves further confirmed the interaction (Fig. 7d and Supplementary Fig. 10). Since there are three major domains on BOP proteins, to understand how they interact with TfALOG3, we further split TfBOP1/2 into different parts and performed Y2H assays (Supplementary Fig. 11). Full-length TfBOP2 or TfBOP1 shows the strongest interaction, and its DUF3420, ANK-repeat and C-terminal domains manifest a weaker interaction (Supplementary Fig. 11).

Since TfALOG3 can physically interact with TfBOP2 in the nucleus, we performed comparative transcriptomic analysis to identify potential downstream targets of these genes (Supplementary Fig. 12 and Supplementary Data 4). Compared with WT, 153 and 85 common genes were down- and upregulated in the two mutants (Supplementary Fig. 12). Consistent with more severe morphological changes, more genes were significantly changed in the *tfbop2-2* mutant. KEGG analysis indicated that most of the downregulated genes were annotated with genetic information processing, carbohydrate metabolism and environmental information processing, while more than half of the upregulated genes belonged to energy metabolism (Supplementary Fig. 12). Among the commonly downregulated genes (Supplementary Data 4), *TfB095441* encodes a LOB domain-containing (LBD) protein and could be an interesting downstream candidate since LBD proteins are thought to play key roles in lateral organ development, such as lateral root formation and leaf development^33^. Other interesting candidates include a class I TCP protein encoded by *TfB077865* and *TfA005723*, which encodes a MYB-like transcription factor, as both the TCP and MYB families have been shown to be involved in a variety of plant developmental processes^34^.

Altogether, these findings provide biochemical evidence for the formation of the BOP-ALOG complex and favour a shared working module of BOP and ALOG factors in various plant lineages.

## Discussion

The formation of corolla tubes has evolved multiple times independently and is considered a developmental innovation of asterids, which include many species with morphologically complex floral structures^35^. Diversification in the corolla tube helps asterid species attract different groups of animal pollinators, which in turn promotes rapid plant speciation^36^. The corolla of *T. fournieri* displays a typical bee pollination syndrome along the proximodistal axis (Supplementary Fig. 1), with a distal lobe region functioning as a billboard and landing platform, an intermediate tube with contrasting carotenoid pigments as nectar guides, and a proximal neck region that helps to protect the nectar. Such proximodistal differentiation, evident in various flowers, is an outstanding example of plant evolution and reproductive success, raising questions about its origin from the perspective of molecular genetics.

In *Torenia*, two paralogues of *BOP* genes exhibit distinct expression patterns, indicating functional divergence after duplication (Fig. 1). *TfBOP1* is expressed in various tissues, including vegetative and floral meristems, and such a pleiotropic expression pattern is similar to those of most previously reported *BOP* genes of core eudicots and monocots^20,23,25,26^, suggesting an ancestral function of *BOP*s. *TfBOP2* displays predominant expression in floral tissues, similar to the tobacco *BOP* gene *NtBOP2*. *NtBOP2* is primarily expressed at the base of the flower where abscission zones are formed, and alteration in its expression causes defects not in the course of corolla growth and development but in corolla abscission^37^. We detected specific expression of *TfBOP2* from stage 6, rather than early stages such as floral meristem or petal primordia, suggesting that it functions after petal initiation (Fig. 1). Consistent with its expression pattern, we observed obvious developmental defects from stage 6 of *TfBOP2-*Cas9 flowers (Fig. 3). The proximal corolla regions of *TfBOP2-*Cas9, including the proximal part of the corolla tube and the proximal corolla neck, become abnormal, which is also evident in the epidermal cell pattern and pigmentation (Figs. 2 and 6). During the floral ontogeny of *T. fournieri*, five petal primordia emerge independently and later fuse together at their bases after stage 6, supporting a characteristic of late sympetaly^11^. We also observed abnormal petal fusion in mutant flowers, favouring the hypothesis that *TfBOP2* simultaneously coordinates proximal corolla differentiation and sympetaly. In spite of these findings, however, we should note that key factors involved in PD patterning are yet to be unveiled for the following two reasons. First, the proximal differentiation of the *TfBOP2-*Cas9 corolla is not completely lost, and we can still observe proximodistal differentiation in *TfBOP2-*Cas9 flowers. Second, organ proximodistal polarity is thought to be established as early as the initiation of petal primordia, and mutation in a key regulator of PD patterning in earlier stages would have resulted in extensive conversion of one region into another.

We investigated possible downstream targets of TfBOP2 through transcriptome sequencing. Genes involved in meristem and boundary formation, epidermis differentiation, carotenoid biosynthesis, cell wall modification, and auxin signalling were significantly changed in the mutant (Fig. 4). Among these genes, we noticed that two *KNOX I* genes, *TfKNAT2* and *TfKNAT6*, were significantly upregulated in the *TfBOP2-*Cas9 mutant (Fig. 4b). There are five KNOX I proteins in *T. fournieri*, and TfKNAT2 and TfKNAT6 are orthologous proteins of Arabidopsis KNAT2 and KNAT6 (Supplementary Fig. 4). In the Arabidopsis *bop1-1* mutant, three *KNOX I* genes, *KNAT1*, *KNAT2* and *KNAT6*, are expressed in an expanded region of the leaf, suggesting that BOP negatively regulates *KNOX I* genes and thus changes the meristematic activity of leaf cells^21^. The repression of *KNOX I* genes at the leaf base may be generated through direct activation of *ASYMMETRIC LEAVES2* (*AS2*), which promotes adaxial leaf polarity and inhibits the expression of *KNOX I* genes, possibly by the formation of a protein dimer with ASYMMETRIC LEAVES1 (AS1)^21^. Interestingly, Arabidopsis BOPs likely differentially regulate *KNOX I* in inflorescences, as *KNAT6* is misexpressed in *BOP1* gain-of-function plants, and such misexpression is required for inflorescence defects^38^. *TfKNAT2* and *TfKNAT6* are upregulated in *TfBOP2* loss-of-function mutants, suggesting that TfBOP2 can negatively regulate *KNOX I* activities in *Torenia*. Numerous studies have demonstrated that KNOX I proteins function in pluripotent cells of meristems as well as differentiating cells of various organs and are vital for primordium initiation, boundary formation and meristem maintenance^39^. Hence, we infer that the development of ectopic boundaries in *TfBOP2-*Cas9 flowers could result from the ectopic expression of *TfKNAT2* and *TfKNAT6*.

In this study, we found that TfBOP2 works together with TfALOG3 in the control of proximal petal differentiation. Either *TfALOG3* or *TfBOP2* is highly expressed in the proximal region of corollas (Fig. 1), suggesting that they may function together as a module regulating corolla development^28^. Although similar defects of the proximal corolla have been observed, their developmental defects are not identical. First, unlike *tfalog3-40*, the distal lobe regions are longer and the whole tubes of the *tfbop2-2* mutant are affected, suggesting that TfBOP2 functions in a broader region than TfALOG3 (Fig. 6). Second, in *tfalog3-40*, the proximal corolla neck is completely lost^28^; however, in *tfbop2-2*, we can still determine a constriction region at the base, although this structure is no longer the same as that in the WT (Fig. 6 and Supplementary Fig. 9). This is reasonable since the expression of TfALOG3, which is responsible for the formation of constriction, was not changed in the *tfbop2-2* mutant. When TfALOG3 is further mutated, the constriction region together with the connected base is expected to be lost, which is evident in a shortened yellow tube of the *tfalog3-40 tfbop2-2* double mutant (Fig. 6). Interestingly, corolla fusion defects in the *tfbop2-2* mutant were largely rescued in the double mutants (Fig. 6), indicating that TfALOG3 may antagonize TfBOP2’s activity in corolla boundary formation. These data suggest that TfBOP2 and TfALOG3 have overlapping roles in regulating the growth of the proximal region of the corolla.

We further demonstrated that TfBOP2 can physically interact with TfALOG3, and in the presence of TfALOG3, TfBOP2 proteins are recruited to the nucleus (Fig. 7). In fact, BOP- and ALOG-like proteins show similar abilities to form BOP–ALOG complexes in other plants. In tomato, three *BOP* genes are redundantly expressed in the vegetative and transition meristem, and by forming different complexes, they act synergistically with TMF, an ALOG family protein, and thus regulate inflorescence architecture and leaf axil patterning^29,30^. *COCHLEATA* (*COCH*), a *BOP1/2* orthologue from *Pisum sativum*, is expressed in various tissues and has pleiotropic roles in multiple developmental processes, including nodule development and lateral organ morphogenesis^31^. COCH interacts with an ALOG protein, SYMMETRIC PETALS 1 (SYP1), resulting in repression of SYP1 degradation^31^. These studies indicate the general existence of BOP-ALOG protein complexes, at least among core eudicots. Based on these findings, we proposed a model of corolla differentiation in the proximal region in *T. fournieri* (Supplementary Fig. 13). The corolla lobe is specified first, followed by the specification of tube; The corolla tube is further differentiated into proximal and distal regions as intercalary growth occurs; The differentiation of proximal corolla tube is controlled by TfBOP2, together with TfALOG3 that specifies the formation of neck region over time (Supplementary Fig. 13).

To summarize, we report a floral-specific BOP from *T. fournieri*, TfBOP2, which acts synergically with TfALOG3 and couples both proximal corolla development and sympetaly. In the future, it will be of interest to investigate whether the BOP-ALOG module has been recruited several times independently during the formation of corolla tubes in distinct lineages. It will also be important to unveil the nature of the molecular agents and mechanisms that pattern the proximodistal axis of petals.

## Methods

### Plant materials and transformation

Plants of *T. fournieri*, *A. thaliana*, and *Nicotiana benthamiana* were maintained at 25 °C under a 16 h light:8 h dark cycle. The Arabidopsis *bop1-3 bop2-1* mutant (CS69935) was obtained from the Arabidopsis Biological Resource Center (ABRC) and generated by crossing *bop1-3* (identified from SALK_012994) with *bop2-1* (identified from SALK_075879). Identification of the *bop1 bop2* double mutant was checked by genotyping and further confirmed by its leaf phenotype (Supplementary Fig. 7).

To carry out plant transformation of *T. fournieri*, leaf discs of *T. fournieri* were collected from sterile plants grown on 1/2 MS culture medium. The CRISPR/Cas9 binary vector was produced according to the published protocol^40^. Specifically, four target sequences with a protospacer adjacent motif (NGG) were designed within the nonconserved regions of TfBOP1/2 using CRISPR-GE (http://skl.scau.edu.cn/). To avoid off-target effect, the candidate target sequences were queried to the *Torenia* genome, ensuring at least 2 bp different from any similar non-target sequences. Transformation of the CRISPR/Cas9 binary vector into WT plants was performed as previously reported^11,28,41^. Briefly, the vectors were introduced into *Agrobacterium tumefaciens* strain EHA105 and transformed into *T. fournieri* using a leaf-disc transformation protocol. Hygromycin-resistant plants were identified by PCR. Genotyping of mutants was carried out by amplifying the target-containing genomic fragments from plants. The PCR products were purified before Sanger sequencing using the sequencing primers. In total, seven hygromycin-resistant lines with similar floral phenotypes were obtained, and the *TfBOP2* genes from two independent lines were sequenced prior to further analysis. Different numbers of nucleotides deletions or insertions in the targeted sites of *TfBOP2* from two independent lines were detected, without any change within *TfBOP1*, supporting the specificity of editing. The primers used here are listed in Supplementary Data 5.

### Phylogenetic and expression analysis

To determine the phylogenetic relationship of BOP proteins in plants, we collected homologous gene sequences from Phytozome (https://phytozome.jgi.doe.gov) and other published genome databases^42,43^. Alignments of amino acid sequences were performed in MEGA X^44^. The maximum-likelihood tree of BOP proteins under 500 bootstrap iterations was also generated by MEGA X. For qRT‒PCR analysis, total RNA and cDNA templates were prepared as reported^28^. Briefly, 1 µg of total RNA was reverse transcribed using the PrimeScript RT Reagent Kit with gDNA Eraser (Takara). qRT‒PCR was performed using a LightCycler 480 Real-Time PCR System (Roche) according to the manual. *β-Actin* (*TfACT3*) was used as a reference gene as previously reported^11,28,45,46^. Data were normalized against *TfACT3* and are summarized from three biological replicates. The primers used here are listed in Supplementary Data 5.

For RNA in situ hybridization, inflorescences were fixed in FAA fixative (10% formalin solution, 5% acetic acid and 50% ethanol). The tissues were then embedded with Paraplast-plus (Sigma‒Aldrich) after treatment with multiple ethanol and xylene solutions. To generate probes, partial cDNA sequences were amplified and cloned into the pTA2 plasmid (TOYOBO) that contains T7 and T3 RNA polymerase promoters for in vitro transcription. The probes were labelled with DIG RNA Labeling Mix (Roche) and purified by LiCl-based precipitation. Hybridization processes were performed as described^47^. Briefly, microtome sections of 10 µm thickness were fixed onto glass slides treated with 0.1% poly-L-lysine. After being deparaffinized and rehydrated, slides were treated with 0.125 mg/ml of proteinase at 37 °C for 10 min. slides were further incubated in 4% paraformaldehyde solution and acetic anhydride solution (0.5% acetic anhydride, 100 mM triethanolamine), each for 10 min, followed by incubation in series of ethanol solutions for dehydration. The hybridization process was performed at 50 °C in hybridization buffer (10 mM Tris-HCl, pH 8, 300 mM NaCl, 10 mM NaH2PO4, 5 mM EDTA, 0.025% Ficoll 400, 0.025% polyvinylpyrrolidone, 0.025% bovine serum albumin, 12.5% dextran sulfate, 0.02% bovine serum albumin, 125 µg/ml yeast tRNA and 50% formamide) with probes overnight. After the hybridization, the slides were washed in SSC (Saline Sodium Citrate), RNase A (0.02 mg/ml) and NTE (10 mM Tris-HCl, pH 7.5, 0.5 M NaCl, and 1 mM EDTA) buffers to remove free probes. For immunological reaction, sections were incubated in DIG buffer (0.1 M Tris-HCl, pH 7.5, 0.15 M NaCl, 1% bovine serum albumin, 0.3% Triton X-100) containing 0.1% Anti-Digoxigenin-AP (Roche) at 25 °C for 2 h. The slides were further washed and stained by NBT/BCIP (4-nitro blue tetrazolium chloride/5-bromo-4-chloro-3-indolyl phosphate) solution (100 mM Tris-HCl pH9.5, 100 mM NaCl, 50 mM MgCl2, 0.15 mg/ml NBT, 0.075 mg/ml BCIP) up to 48 h according to staining degree. To examine signals, slides were mounted by Neutral Balsam prior to observation. The primers used here are listed in Supplementary Data 5.

### Microscopy

To compare the floral development of different plants, floral buds of different stages were collected and dissected using fine tweezers under a stereomicroscope. To observe corolla epidermal cells, mature corollas from various genotypes were cut by a razor blade. Floral buds and corollas were rapidly covered by Imprint^TM^ II Garant (3 M ESPE) impression materials and filled with 2-Ton Epoxy adhesive (Devcon Ltd.) to produce the epoxy replica as previously reported^9^. The epoxy replicas were then directly observed under a Keyence VHX-D510 microscope. Images were measured with Adobe Photoshop CS6 according to the measurement protocol (https://helpx.adobe.com/photoshop/using/measurement.html), and statistical analysis was performed in GraphPad Prism 8.

### Transcriptomic analysis

Tube regions of stage 10 corollas were collected from the WT, *tfbop2-2*, *tfalog3-40* plants. We pooled tissues from ten flowers as one biological replicate, and three biological replicates for each genotype were harvested. Total RNA was extracted using the RNAqueous-Micro kit and Plant RNA Isolation Aid according to the manual (Ambion). The cDNA libraries were constructed using the TruSeq RNA Sample Preparation kit (Illumina). Sequencing, assembly and annotation of contigs were carried out as previously reported^48^. Specifically, the libraries were sequenced with an Illumina Genome Analyser IIx Sequencer with a single-end module (Illumina), and clean reads were trimmed using bcl2fastq (Illumina). Short reads (<50 bp) were discarded prior to mapping (Supplementary Data 6). In total, 121 million sequencing reads (25.2 Gb) were generated, and 87.74% of them were mapped to the *Torenia* database (http://dandelion.liveholonics.com/torenia/). Hierarchical clustering of different samples favours high reproducibility among the biological replicates (Supplementary Fig. 14). The relative amount of each transcript was quantified with Salmon, and differential expression analysis was conducted using the EdgeR package. Significant DEGs were identified as those with FDR-adjusted *p* value <0.0001 and |LogFC|> 1. Gene ontology annotations were performed by the Blast2GO program.

### Protein localization and bimolecular fluorescence complementation (BiFC) assay

To generate the constructs for subcellular localization, the coding sequence for Venus protein was fused to the sequence encoding the C-terminus of TfALOG3, and the coding sequence of mRuby2 was fused to the sequence encoding the C-terminus of TfBOP2. The expression of those chimeric genes was driven by the CaM35S promoter. To produce vectors for the BiFC assay, coding sequences of TfALOG3 and TfBOP2 were ligated into split YFP vectors driven by the CaMV35S promoter. The constructed plasmids were transformed into *Agrobacterium tumefaciens* EHA105 and then transfected into tobacco leaves through infiltration. After two days, tobacco leaves were placed on slides with the abaxial side up, and the fluorescence was examined under appropriate channels of a Zeiss 7 DUO NLO confocal laser scanning microscope. For DAPI (4’,6-diamidino-2-phenylindole) staining, tobacco leaves were incubated in 10 μM DAPI solution for 10 min before observation. The DAPI, yellow fluorescent protein (YFP/Venus) and monomeric red fluorescent protein (mRuby2) were excited by 405, 515 and 559 nm diode lasers, respectively. Maximum-intensity projections were used for imaging with pseudocolour (blue for DAPI; yellow for YFP; green for Venus and magenta for mRuby2). The primers used here are listed in Supplementary Data 5.

### Yeast two-hybrid (Y2H) assay

Yeast two-hybrid assays were conducted according to the manufacturer’s manual (Takara). Full-length or truncated TfALOG3 and TfBOPs were amplified and cloned into pGADT7 and/or pGBKT7 vectors. After sequencing, the correct plasmids were cotransformed into yeast strain AH109, and the clones were screened on SD/-Trp/-Leu and SD/-Trp/-Leu/-His/-Ade plates supplemented with X-α-gal (80 mg/ml). The primers used here are listed in Supplementary Data 5.

### Coimmunoprecipitation (CoIP) assay

For Co-IP using the tobacco transient expression system, full-length coding sequences of *TfALOG3* and *TfBOP2* were amplified from *T. fournieri* cDNA and cloned into the BamHI sites of the pHB-HA or pHB-FLAG vector^49^. CoIP assays were conducted as described^42^. Briefly, a mixture of *Agrobacterium tumefaciens* strain *GV3101* with HA and FLAG plasmids was infiltrated into tobacco leaves. Forty-eight hours after infiltration, leaves were collected and homogenized in lysis buffer (50 mM HEPES pH 7.5, 150 mM NaCl, 10 mM EDTA pH 8.0, 1% Triton X-100, 0.2% Nonidet P-40, 10% glycerol, 2 mM DTT, 1X Complete Protease Inhibitor Cocktail (Roche)). Extracts were centrifuged, filtered and incubated with anti-HA beads (Sigma‒Aldrich, cat. no A2095). After 3 hr of incubation, the beads were centrifuged and washed with wash buffer (50 mM HEPES pH 7.5, 150 mM NaCl, 10 mM EDTA pH 8.0, 0.1% Nonidet P-40, 10% glycerol, 2 mM DTT) prior to sodium dodecyl sulfate‒polyacrylamide gel electrophoresis (SDS–PAGE). Western blotting was carried out using anti-HA (Sigma‒Aldrich, cat. no. H6533, 1:5000) and anti-FLAG antibodies (Sigma‒Aldrich, cat. no. A8592, 1:5000). The primers used here are listed in Supplementary Data 5.

### Reporting summary

Further information on research design is available in the Nature Portfolio Reporting Summary linked to this article.

### Supplementary information

Supplementary Information

Description of Additional Supplementary Files

Supplementary Dataset 1

Supplementary Dataset 2

Supplementary Dataset 3

Supplementary Dataset 4

Supplementary Dataset 5

Supplementary Dataset 6

Reporting Summary

### Source data

Source Data

## Data Availability

Data from this study are available upon request to the corresponding author. The raw RNA-sequencing data were deposited in the Sequence Read Archive (Accession: PRJNA938788). Source data are provided with this paper.

## References

[1] Sablowski R (2015). Control of patterning, growth, and differentiation by floral organ identity genes. J. Exp. Bot..

[2] Moyroud E, Glover BJ (2017). The evolution of diverse floral morphologies. Curr. Biol..

[3] Fernandez-Mazuecos M, Glover BJ (2017). The evo-devo of plant speciation. Nat. Ecol. Evol..

[4] Darwin, C. R. *On The Origins of Species by Means of Natural Selection* (Murray, 1859).

[5] Chanderbali, A. S. et al. Evolution of floral diversity: genomics, genes and gamma. *Philos. Trans. R. Soc. Lond. B Biol. Sci.*10.1098/rstb.2015.0509 (2017). 10.1098/rstb.2015.0509 PMC5182423 27994132

[6] Shan H (2019). Developmental mechanisms involved in the diversification of flowers. Nat. Plants.

[7] Coen ES, Meyerowitz EM (1991). The war of the whorls genetic interactions controlling flower development. Nature.

[8] Su, S. et al. An *AGAMOUS*-like factor is associated with the origin of two domesticated varieties in *Cymbidium sinense* (Orchidaceae). *Hortic. Res*. **5**, 48 (2018). 10.1038/s41438-018-0052-z PMC6119200 30181888

[9] Su S (2018). Transcriptome-wide analysis reveals the origin of peloria in Chinese *Cymbidium* (*Cymbidium sinense*). Plant Cell Physiol..

[10] Feng C (2012). Evolution of bract development and B-class MADS box gene expression in petaloid bracts of *Cornus* s. l. (Cornaceae). N. Phytol..

[11] Su S (2017). The CYCLOIDEA-RADIALIS module regulates petal shape and pigmentation, leading to bilateral corolla symmetry in *Torenia fournieri* (Linderniaceae). N. Phytol..

[12] Luo D (1995). Origin of floral asymmetry in *Antirrhinum*. Nature.

[13] Luo D (1999). Control of organ asymmetry in flowers of *Antirrhinum*. Cell.

[14] Satterlee, J. W. & Scanlon, M. J. Coordination of leaf development across developmental axes. *Plants* **8**, 433 (2019). 10.3390/plants8100433 PMC6843618 31652517

[15] Sauret-Gueto S (2013). *JAGGED* controls *Arabidopsis* petal growth and shape by interacting with a divergent polarity field. PLoS Biol..

[16] Ohno CK (2004). The *Arabidopsis JAGGED* gene encodes a zinc finger protein that promotes leaf tissue development. Development.

[17] Crawford B (2004). *CINCINNATA* controls both cell differentiation and growth in petal lobes and leaves of *Antirrhinum*. Plant Physiol..

[18] Julio R (2009). Distal expression of *knotted1* in maize leaves leads to reestablishment of proximal/distal patterning and leaf dissection. Plant Physiol..

[19] Hepworth SR (2005). BLADE-ON-PETIOLE-dependent signaling controls leaf and floral patterning in *Arabidopsis*. Plant Cell.

[20] Norberg M (2005). The *BLADE ON PETIOLE* genes act redundantly to control the growth and development of lateral organs. Development.

[21] Ha CM (2003). The *BLADE-ON-PETIOLE 1* gene controls leaf pattern formation through the modulation of meristematic activity in *Arabidopsis*. Development.

[22] Chahtane H (2018). LEAFY activity is post-transcriptionally regulated by BLADE ON PETIOLE2 and CULLIN3 in *Arabidopsis*. N. Phytol..

[23] Couzigou JM (2012). *NODULE ROOT* and *COCHLEATA* maintain nodule development and are legume orthologs of *Arabidopsis BLADE-ON-PETIOLE* genes. Plant Cell.

[24] Magne K (2018). *Lotus japonicus NOOT-BOP-COCH-LIKE1* is essential for nodule, nectary, leaf and flower development. Plant J..

[25] Toriba T (2019). *BLADE-ON-PETIOLE* genes temporally and developmentally regulate the sheath to blade ratio of rice leaves. Nat. Commun..

[26] Tavakol E (2015). The barley *Uniculme4* gene encodes a BLADE-ON-PETIOLE-like protein that controls tillering and leaf patterning. Plant Physiol..

[27] Khan M, Xu H, Hepworth SR (2014). BLADE-ON-PETIOLE genes: setting boundaries in development and defense. Plant Sci..

[28] Xiao W (2019). A homolog of the ALOG family controls corolla tube differentiation in *Torenia fournieri*. Development.

[29] Xu C (2016). Control of inflorescence architecture in tomato by BTB/POZ transcriptional regulators. Genes Dev..

[30] Izhaki A (2018). The tomato *BLADE ON PETIOLE* and *TERMINATING FLOWER* regulate leaf axil patterning along the proximal-distal axes. Front. Plant Sci..

[31] He L (2020). *SYMMETRIC PETALS 1* encodes an alog domain protein that controls floral organ internal asymmetry in Pea (*Pisum sativum* L.). Int J. Mol. Sci..

[32] Xiao W (2018). Evolution of ALOG gene family suggests various roles in establishing plant architecture of *Torenia fournieri*. BMC Plant Biol..

[33] Zhang YW (2020). Phylogeny and functions of LOB domain proteins in plants. Int. J. Mol. Sci..

[34] Corley SB (2005). Floral asymmetry involves an interplay between TCP and MYB transcription factors in *Antirrhinum*. Proc. Natl Acad. Sci. USA.

[35] Phillips HR, Landis JB, Specht CD (2020). Revisiting floral fusion: the evolution and molecular basis of a developmental innovation. J. Exp. Bot..

[36] Ding B (2020). Developmental genetics of corolla tube formation: role of the tasiRNA-*ARF* pathway and a conceptual model. Plant Cell.

[37] Wu XM (2012). The tobacco *BLADE-ON-PETIOLE2* gene mediates differentiation of the corolla abscission zone by controlling longitudinal cell expansion. Plant Physiol..

[38] Khan M (2012). Antagonistic interaction of BLADE-ON-PETIOLE1 and 2 with BREVIPEDICELLUS and PENNYWISE regulates *Arabidopsis* inflorescence architecture. Plant Physiol..

[39] Hay A, Tsiantis M (2010). KNOX genes: versatile regulators of plant development and diversity. Development.

[40] Ma XL (2015). A robust CRISPR/Cas9 system for convenient, high-efficiency multiplex genome editing in monocot and dicot plants. Mol. Plant.

[41] Aida R (2012). A protocol for transformation of *Torenia*. Methods Mol. Biol..

[42] Xu Z (2016). Transcriptional and post-transcriptional modulation of SQU and KEW activities in the control of dorsal-ventral asymmetric flower development in *Lotus japonicus*. Mol. Plant.

[43] VanBuren R (2018). Desiccation tolerance evolved through gene duplication and network rewiring in *Lindernia*. Plant Cell.

[44] Kumar S (2018). MEGA X: molecular evolutionary genetics analysis across computing platforms. Mol. Biol. Evol..

[45] Shimoda T (2012). The effect of genetically enriched (*E*)-β-ocimene and the role of floral scent in the attraction of the predatory mite *Phytoseiulus persimilis* to spider mite-induced volatile blends of torenia. N. Phytol..

[46] Sasaki K (2012). Mutation in *Torenia fournieri* Lind. UFO homolog confers loss of TfLFY interaction and results in a petal to sepal transformation. Plant J..

[47] Coen ES (1990). *Floricaula*: a homeotic gene required for flower development in *Antirrhinum majus*. Cell.

[48] Okuda S (2013). Acquisition of LURE-binding activity at the pollen tube tip of *Torenia fournieri*. Mol. Plant.

[49] Liu YC (2018). LjCOCH interplays with LjAPP1 to maintain the nodule development in *Lotus japonicus*. Plant Growth Regul..

