# Supplementary material for: A BLADE-ON-PETIOLE orthologue regulates corolla differentiation in the proximal region in Torenia fournieri
Source: Nat Commun. 2023 Aug 8;14:4763. doi: 10.1038/s41467-023-40399-3 (PMC10409793; doi:10.1038/s41467-023-40399-3)
Supplement: Supplementary file 1 — Supplementary Information [file 41467_2023_40399_MOESM1_ESM.pdf]

Supplementary Figure 1

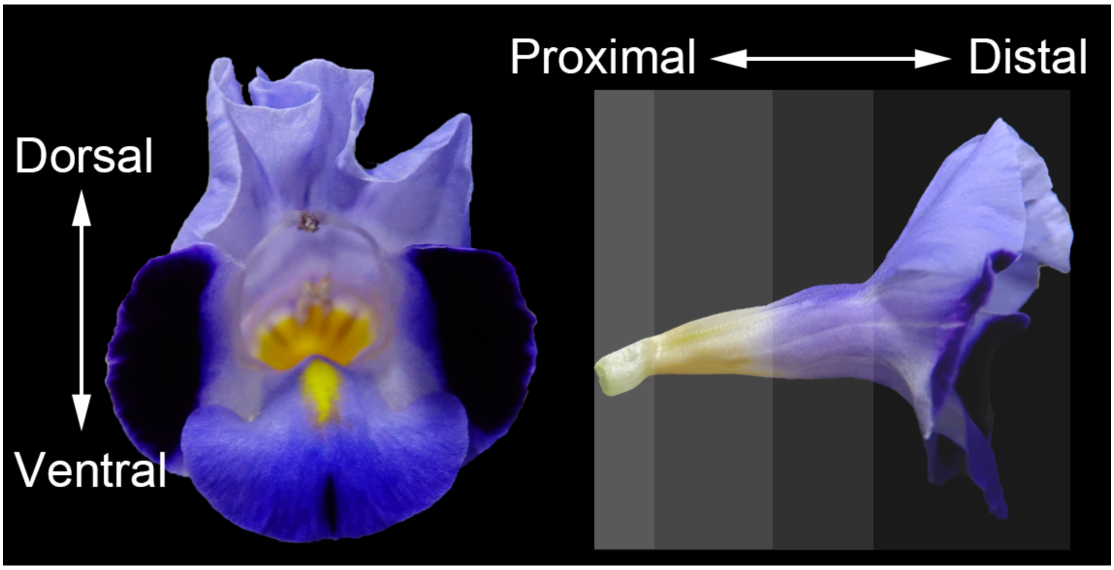

Supplementary Figure 1. Dorsoventral and proximodistal axes of a mature *T. fournieri* flower. Sub-regions along the petal proximodistal axis are highlighted. The distal part of petals forms a lobe region, with violet pigments on lateral and ventral petal lobes; a middle corolla region forms a conical tube, subdivided into a purple distal part and a yellow proximal; and a white corolla base specified into an inflated neck region.

Supplementary Figure 2

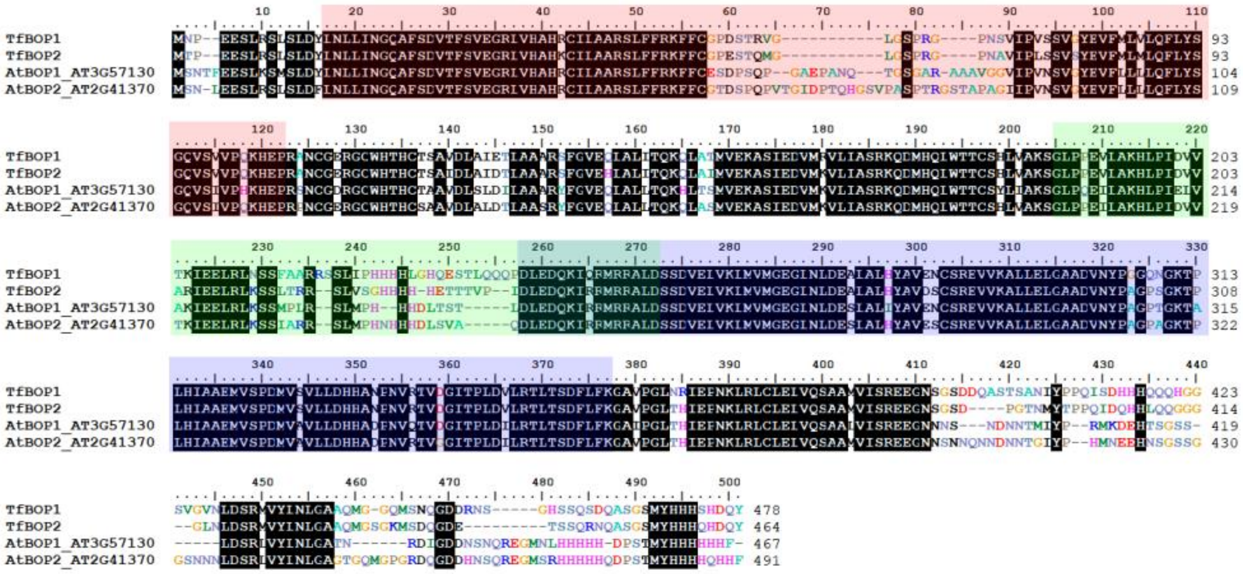

Supplementary Figure 2 Alignments of TfBOP1/2 and AtBOP1/2 proteins. BTB/POZ domain (Pfam: 00651), DUF3420 domain (Pfam: 11900) and ankryin (ANK) domain (Pfam: 00023) are highlighted by red, green and blue boxes, respectively. DUF3420 and ANK domains are partially overlapped.

Supplementary Figure 3

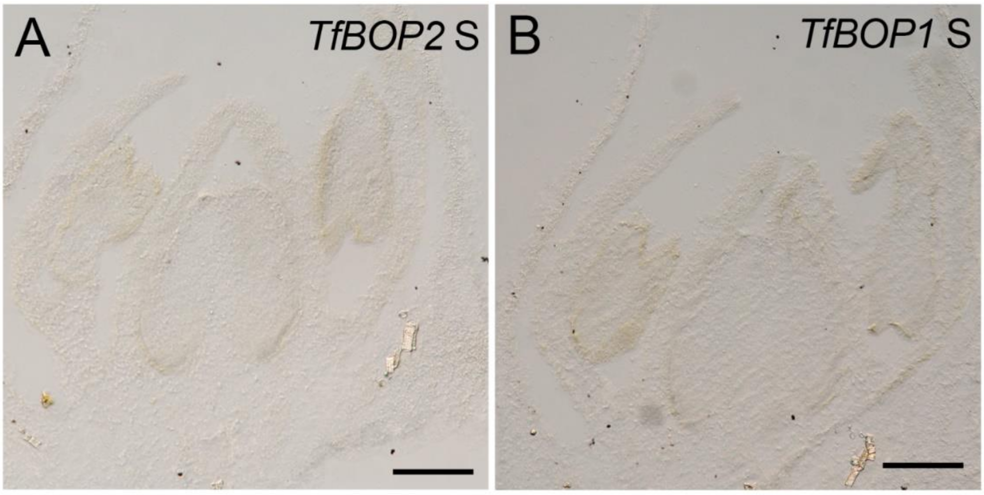

Supplementary Figure 3. RNA *in situ* hybridization of *TfBOP2* (A) and *TfBOP1* (B) in stage 8 flower buds of wild-type using sense probes. Scale bars: 100 μm. Similar results were obtained from three independent experiments.

Supplementary Figure 4

```
>TfBOP2
ATGACCCCCGAAGAGTCGCTGAGATCCCTCTCCTTGGACTACCTCAACCTGTTAATCAACGGTCAGGCCTTCAGCGACGTCACCTT
CAGCGTGGAGGGGCGTTTGGTCCACGCGCACAAGTGCATACTCGCGGCACGGAGCTTGTTCTTCAGGAAATTCTTCTGCGGGCC
CGAGAGCACGCAGATGGGCCTGGGCTCGCCGCGCGGGCCCAACGCCGTGATACCGCTGAGCTCGGTGAGCTACGAGGTCTTCAT
GCTCATGCTTCAGTTCTTGTACAGCGGGCAAGTCTCGGTTGTGCCTCAGAAGCACGAGCCGAGGGCTAACTGCGGAGAGCGAGG
ATGCTGGCACACGCATTGCACGTCCGCCATTGATCTCGCCATCGACACACTCGCAGCAGCCAGATCTTTCGGTGTTGAGCACCTCG
CTTTGATCACgcaggtatatctatatatatatatatatatatatcaagaatatataattactctctctaataattttccgatccatattatttacaagtaagtaattaaca
actttttgtttagaaaagatcgatgagccaaagatttatttacaccggccatatctaaaaaataaaaaaataaaaataaaatcttggaccttttgggttttctgctgatata
aaaatgttagtggggttattgtcatctacgtcgtcttatatctctctttttacttgacaagtttatgtctatatgttgcagtcttctgttttctatttatgtgtaataacgaacggg
ctttgatttcgattctccaacactttcttattataaaacccaacctttgtgctatgctatgatggatgatatgatgaagaatcccatttcatgtaacgttgattgtgtagagcga
aatgaggtttcttttactattttctttttcattaactgtggaaactttctacatttggtttattcgctgtgtctggtcaagtcttgtcttccacaatgacctcatcgtctcatcagatc
ttttgaaagtgccttcacattccccaatttgttcgatatcaaatcttacctttcatgctcatattgttactcacatttaattcatttccagaaacataagataatctgttcgatcag
ttttttttttaactataaatataaattaatcatcttctctttctattatcaatatttcgttatatgctgtgtgtgaattctgttatataatttttgatgtgtcaggtgttgcatgcta
tgatttcaagaaaaataagcggagatatcaaattgggtgactaactagctgctattattattagtctcatacacattcgcatattgaaacagctatgattcacacgctgatttt
gcatgcgtgttctattgcaaaactaattaatttcaggatcggatttacttttacggtgtgttttcatgcattagtgtgctaagtttggttgatatataattttcaaatattctctaat
acaattatttatgcgtatgatgaaaaaggatgcaatcaaatgcagtaaccttaaattgaactagtacggaaataccgctcgatctatatatatctgctattataatgttactt
ggcgatattggttattgcagactaattaatgctcagtaattcatcagctaattagttatacatacgcaataataattgttGCAGAAGCAACTGGCAATCATGGTA
GAGAAGGCATCGATCGAGGATGTAATGAAAGTCCTCATTGCCTCCCGAAAACAAGACATGCACCAGCTATGGACGACATGTTCCC
ACCTCGTCGCAAAATCCGGCCTCCCACCCGAAGTCTTAGCCAAACACCTCCCAATCGACGTCGTCGCGAGAATCGAAGAGCTCCG
TCTGAAATCATCCCTCACCCGCCGCTCCCTAGTCTCCGGCCACCACCACCACCACGAAACAACCACCGTCCCGATCGATCTCGAAG
ACCAAAAGATACGTAGGATGAGACGAGCCCTCGACTCCTCGGACGTCGAACTCGTAAAGCTCATGGTCATGGGCGAAGGCCTAAA
CCTCGATGAGGCGTTGGCTTTGCACTACGCCGTTGACAGCTGCAGCAGAGAAGTGGTGAAAGCCCTACTCGAACTCGGAGCGGC
CGATGTGAACTACCCGGCGGGCCCCTCTGGAAAAACCCCACTTCACATAGCGGCGGAGATGGTGTCTCCGGACATGGTCTCCGTT
CTTTTGGATCACCATGCAAACCCTAATGTAAGAACGGTCGATGGGATCACGCCATTGGACGTGTTAAGAACCCTAACCTCGGATTT
CCTGTTCAAAGGGGCTGTCCCGGGCCTGACGCACATCGAGCCTAACAAGCTGAGGCTCTGTCTCGAACTCGTTCAATCTGCGGCG
ATGGTGATCTCGAGAGAAGAAGGGAACAGTGGGAGCGATCCGGGGACAAATATGTATACGCCGCCGCAAATCGATCAGCATCATC
TTCAACAAGGTGGTGGTGGGCTTAATCTTGATTCGAGAATGGTGTATTTGAACCTCGGTGCTGCGCAGATGGGATCGGGGAAGAT
GAGCGATCAAGGCGACGAGACGAGCAGCCAAAGAAATCAAGCTTCGGGTTCAATGTACCATCATCATCAGCACGATCAGTATTGA
```

```
WT: ...TCGAAGACCAAAAGATACGTAGG...AACTACCCGGCGGGCCC--CTCTGG...GGAACAGTGGGAGCGAT-CCGGGG...
2# : ...TCGAAGACCAAAAG-----CGTAGG...AACTACCCGGCGGGCCCTCTCTGG...GGAACAGTGGGAGCGATTCCGGGG...
5# : ...TCGAAGACCAAAA-------CGTAGG...AACTACCCGGCGGGCCCTCTCTGG...GGAACAGTGGGAGCGAT-CCGGGG...
```

Supplementary Figure 4. CRISPR/Cas9 genome editing in this study. Upper panel: The genomic DNA sequence of *TfBOP2*. The black and red characters represent exons and introns, respectively; PAMs (Protospacer Adjacent Motifs) and different CRISPR/Cas9 targets are highlighted in blue. Lower panel: Mutations in the WT and two *TfBOP2*-Cas9 plants analyzed in this study.

Supplementary Figure 5

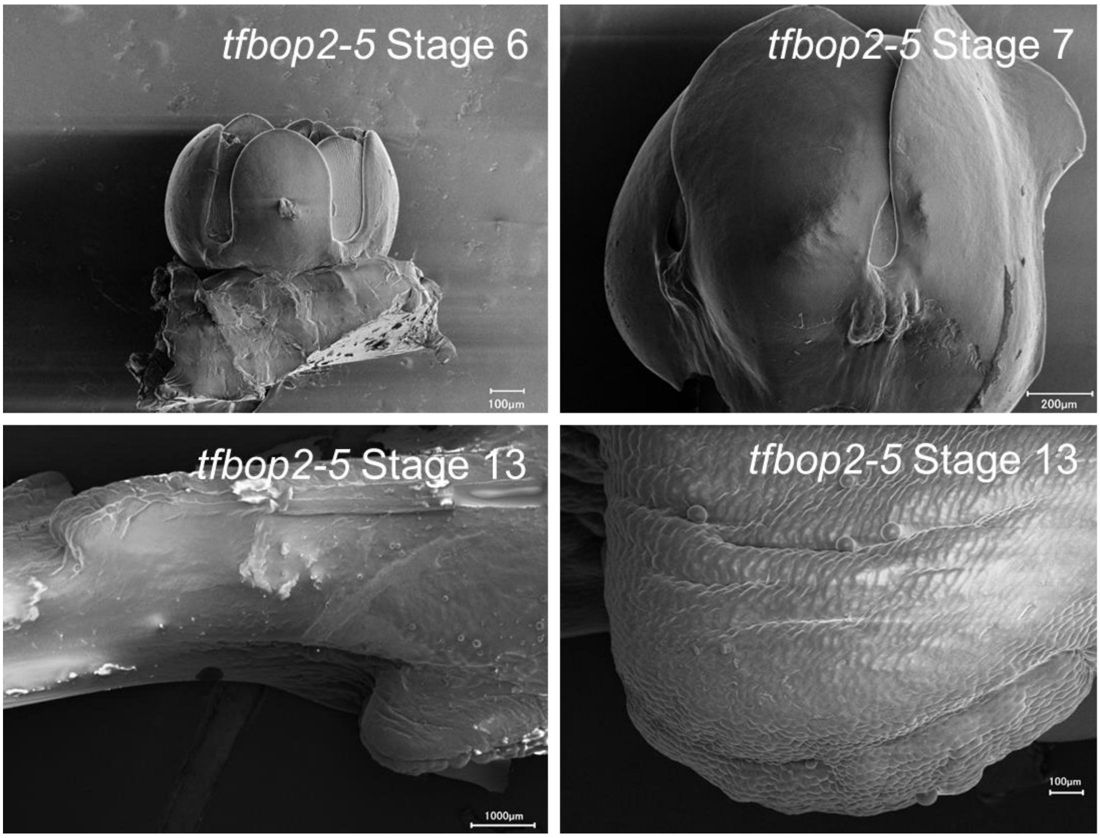

Supplementary Figure 5. Floral organogenesis of TfBOP2-Cas9 5# plant observed by a scanning electron microscopy. Similar results were obtained from three biological replicates.

Supplementary Figure 6

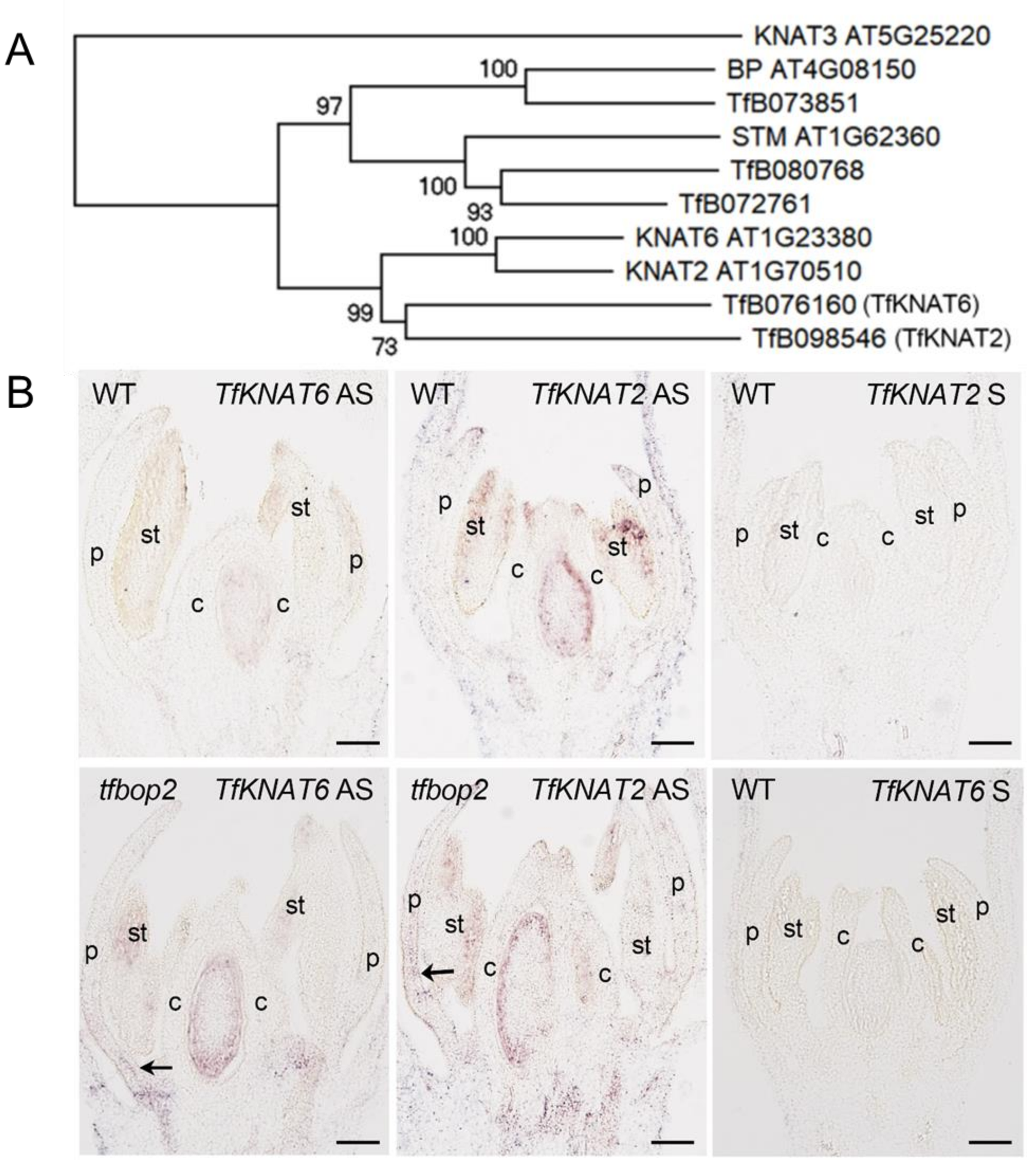

Supplementary Figure 6. Phylogenetic and expression analysis of KNOXs. (A) Neighbor-joining tree for the KNOX transcription factors. The bootstrap value (percentage, based on 1000) is marked at each node, and the accession numbers are indicated as the suffix of each sequence. KNAT3 was chosen as an outgroup. (B) RNA *in situ* hybridization of *TfKNAT2* and *TfKNAT6* in stage 8 flower buds of wild-type and *tfbop2* plants using antisense (AS) or sense (S) probes. p, petal; st, stamen; c, carpel; Ectopic expression of two genes are indicated by black arrows; Scale bars: 100 µm. Similar results were obtained from three independent experiments.

Supplementary Figure 7

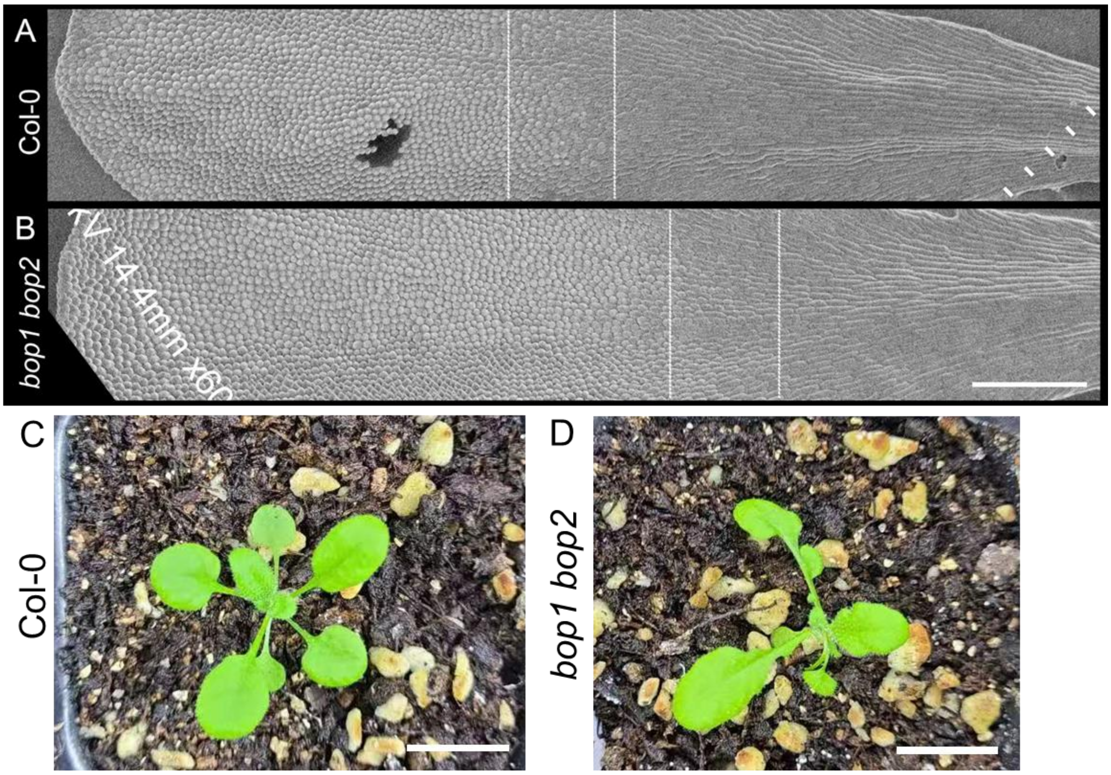

Supplementary Figure 7. Petal epidermal and leaf morphologies of wild-type (A and C) and *bop1 bop2* (B and D) mutant of *Arabidopsis thaliana*. Similar results were obtained from three biological replicates. White lines indicate the proximal claw (right), intermediate (middle) and distal blade (left) regions, respectively; Scale bar: 100 µm. (C-D) 3-week-old plants of wild-type (C) and *bop1 bop2* (D) mutant; Scale bars: 1 cm.

Supplementary Figure 8

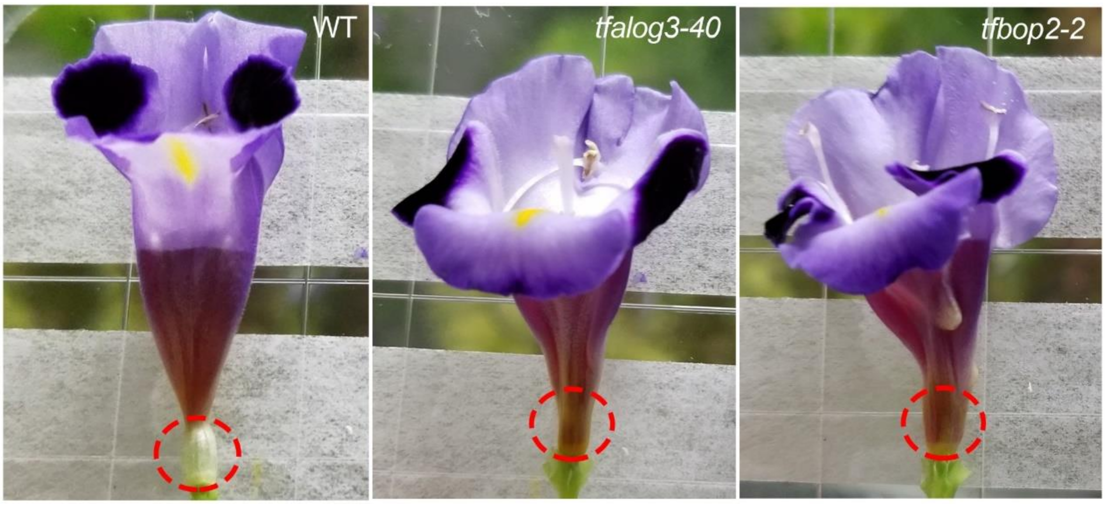

Supplementary Figure 8. Application of 200 µl water containing 0.1% Safranin O to different flowers. The red dashed circle indicates the corolla bottom. Colored water easily entered the nectary of *tfalog3-40* or *tfbop2-2* flowers compared with the WT flower.

Supplementary Figure 9

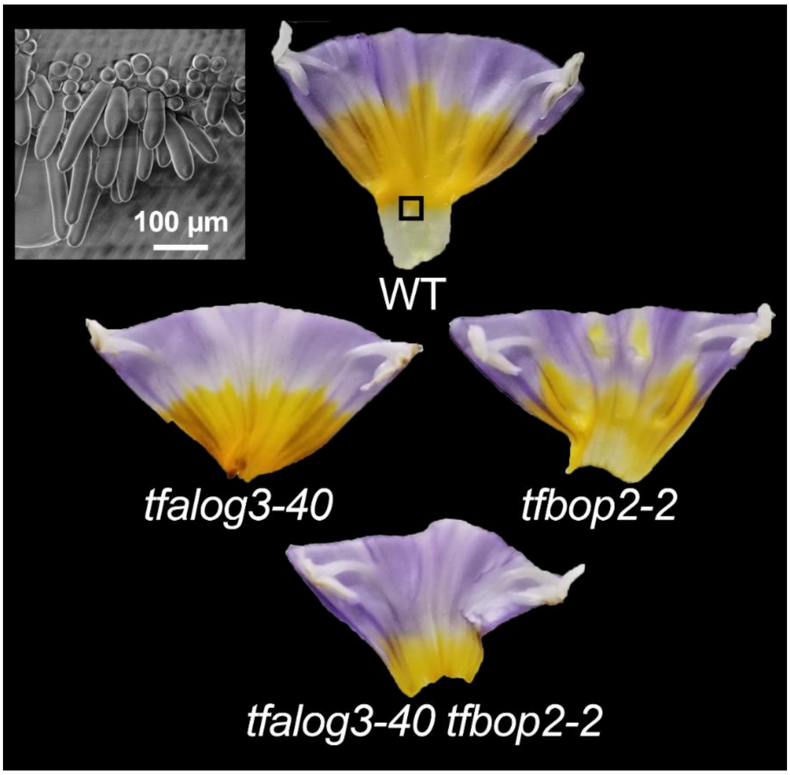

Supplementary Figure 9. Proximal tube regions of WT, *tfalog3-40*, *tfbop2-2* and *tfalog3-40 tfbop2-2* mutants. An SEM photo marked by a black box indicate hairs in the neck region of WT. Both single and double mutants lack such hairs. Similar results were obtained from three biological replicates.

Supplementary Figure 10

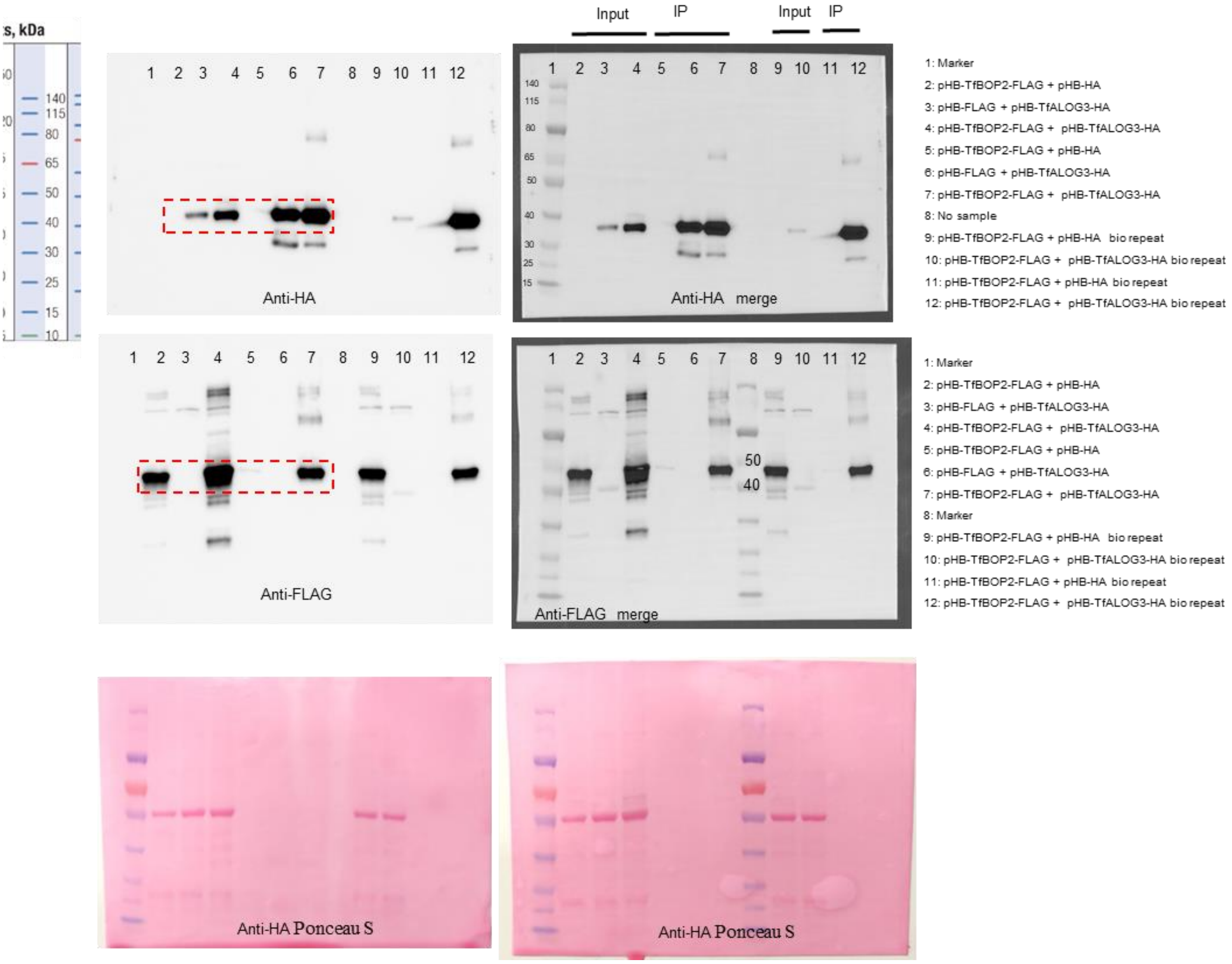

Supplementary Figure 10. Co-immunoprecipitation showing interaction of TfBOP2 with TfALOG3. TfALOG3-HA was co-immunoprecipitated with anti-HA beads from tobacco leaves that co-expressed TfBOP2-FLAG and TfALOG3-HA. Ponceau S staining of Rubisco was shown as a loading control.

Supplementary Figure 11

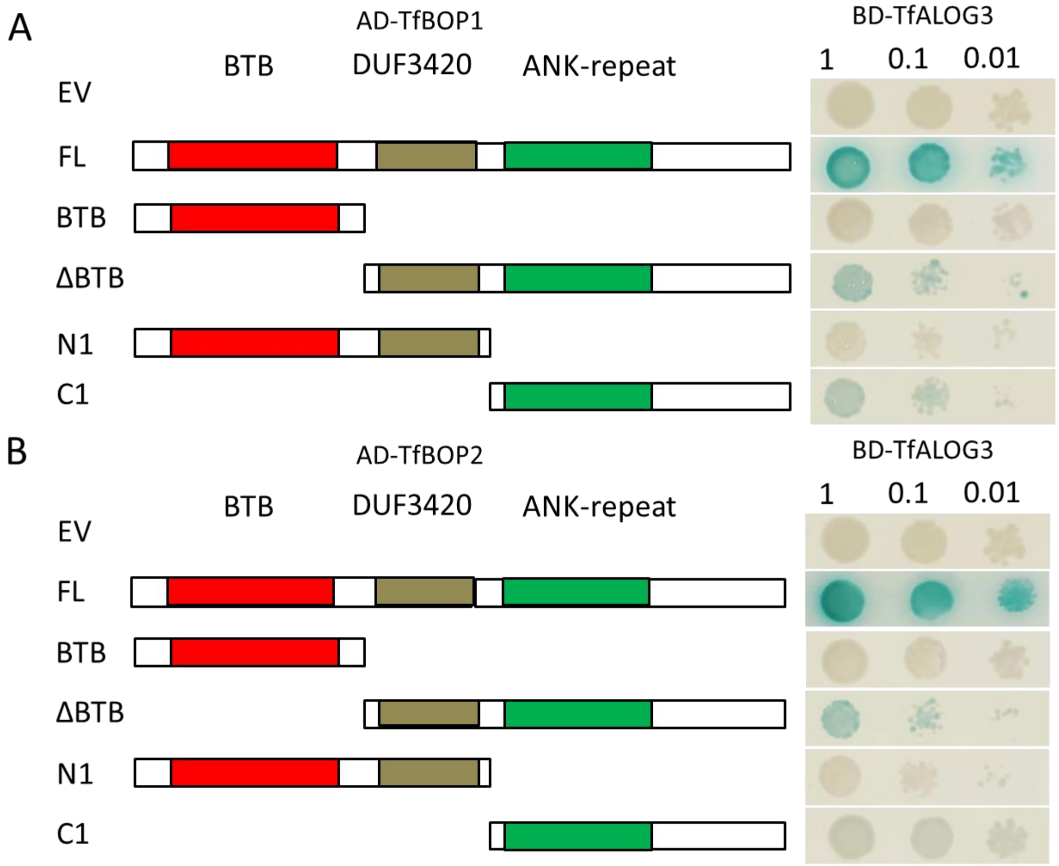

Supplementary Figure 11. Yeast two-hybrid assays using SD-LW/X-Gal media (SD medium minus Trp, Leu and supplemented with X-Gal). Results show that TfBOP1 (A) and TfBOP2 (B) interacts with TfALOG3 partially through the DUF3420, ANK-repeat and C-terminal domains.

Supplementary Figure 12

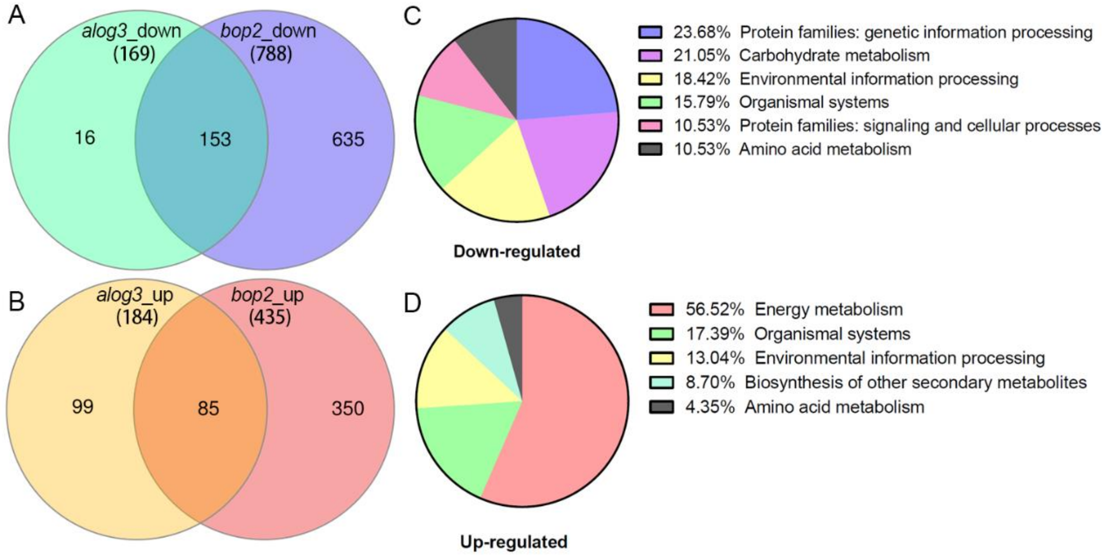

Supplementary Figure 12. Comparative transcriptomic analysis of WT and mutants. (A) Venn diagram showing the number of overlapping down-regulated genes. (B) Venn diagram showing the number of overlapping up-regulated genes. (C) Pie chart of gene ontology (GO) analysis based on overlapping down-regulated genes. (D) Pie chart of GO analysis based on overlapping up-regulated genes.

Supplementary Figure 13

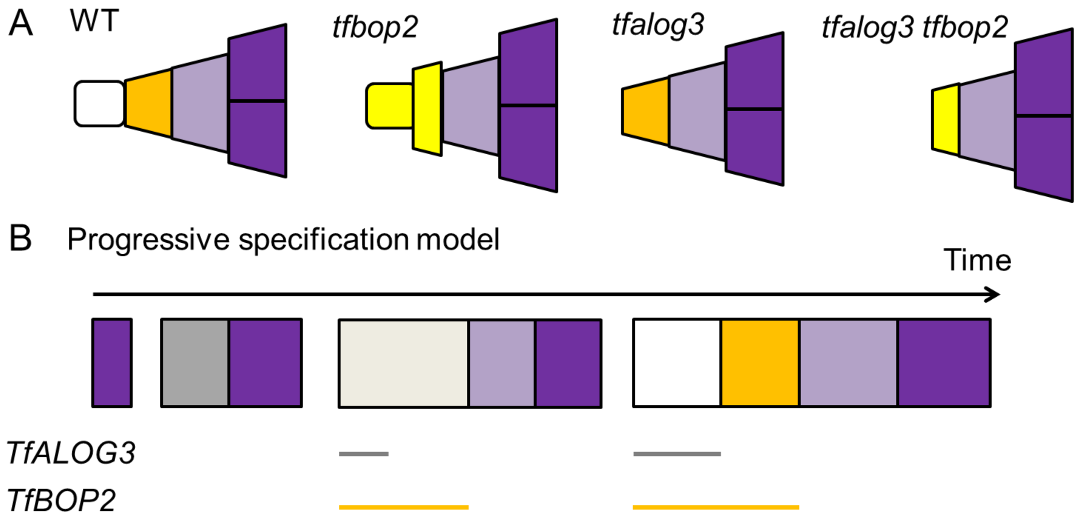

Supplementary Figure 13. A model of corolla differentiation in the proximal region in *T. fournieri*. (A) Phenotypic changes manifested in WT and mutants. Different colors represent different regions. (B) A progressive specification model of corolla PD patterning in *T. fournieri*; Expression patterns of TfALOG3 (gray bars) and TfBOP2 (orange bars) are shown below. The corolla lobe (dark purple block) is specified first, followed by the specification of tube by unknown factors (dark gray block). The corolla tube is further differentiated into proximal (light gray block) and distal (light purple block) regions as growth occurs. The differentiation of proximal corolla tube (white and orange blocks) is controlled by TfBOP2, together with TfALOG3 that specifies the formation of neck region (white block) over time.

Supplementary Figure 14

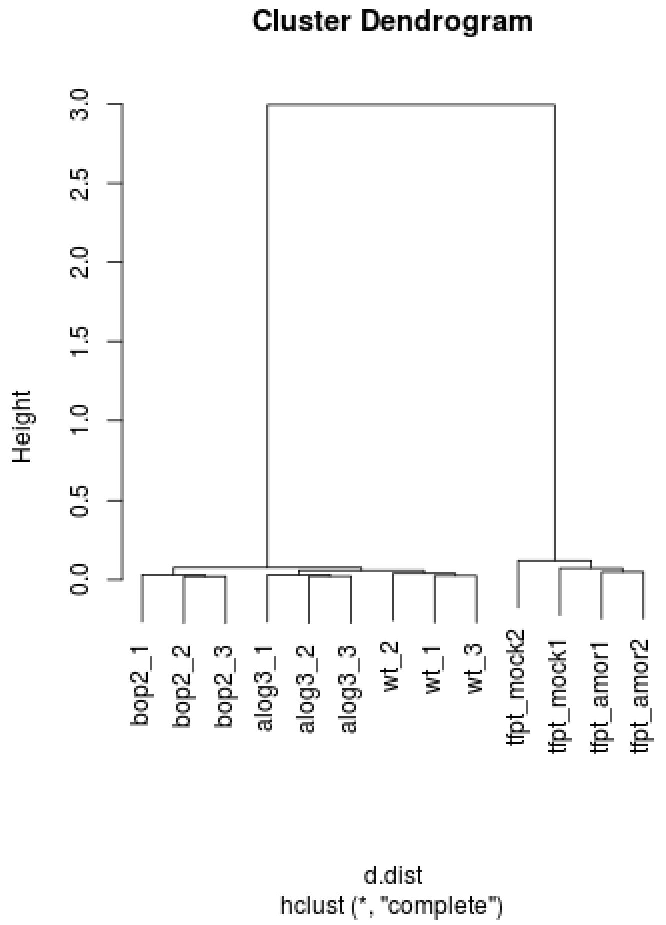

Supplementary Figure 14. A cluster of RNA-seq samples based on the expression pattern of contigs. RNA-seq of pollen tube (tfpt) are separated with corollas of WT (wt), *TfBOP2*-Cas9 (bop2) and*TfALOG3*-Cas9 (alog3).
