# Supplementary material for: A BLADE-ON-PETIOLE orthologue regulates corolla differentiation in the proximal region in Torenia fournieri
Source: Nat Commun. 2023 Aug 8;14:4763. doi: 10.1038/s41467-023-40399-3 (PMC10409793; doi:10.1038/s41467-023-40399-3)
Supplement: Supplementary file 2 — Description of Additional Supplementary Files [file 41467_2023_40399_MOESM2_ESM.pdf]

Description of Additional Supplementary Files:

Supplementary Data 1. Differential gene expression analysis between WT and tfbop2-2 mutants. Differential expression analysis of all genes conducted using the EdgeR package, wt_1/2/3 and bop2_1/2/3 refer to three biological replicates of WT and tfbop2-2 samples. Two-sided P values were used and adjusted for multiple comparison testing (FDR-adjusted)

Supplementary Data 2. Gene Ontology (GO) terms significantly changed in tfbop2-2 mutants compared with WT. Two-sided P values were used and adjusted for multiple comparison testing (FDR-adjusted)

Supplementary Data 3. Differentially expressed genes for heatmap analysis. wt_1/2/3 and bop2_1/2/3 refer to three biological replicates of WT and tfbop2-2 samples. Two-sided P values were used and adjusted for multiple comparison testing (FDR-adjusted).

Supplementary Data 4. Commonly up-regulated and down-regulated genes in WT, tfbop2-2 and tfalog3-40. Significantly down-regulated DEGs identified as those with adjusted p-value <0.0001 and LogFC <-1, wt_1/2/3, alog3_1/2/3 and bop2_1/2/3 refer to three biological replicates of WT, tfalog3-40 and tfbop2-2 samples. Two-sided P values were used and adjusted for multiple comparison testing (FDRadjusted).

Supplementary Data 5. Primers used in this study. Primer names, sequences and purposes were listed.

Supplementary Data 6. Summary of mapping.
